# Genome-wide analysis identifies susceptibility loci for heart failure and nonischemic cardiomyopathy subtype in the East Asian populations

**DOI:** 10.1371/journal.pgen.1011897

**Published:** 2025-10-27

**Authors:** Yi Han, Yun Hong, Yan Gao, Jiapeng Lu, Bowang Chen, Lihua Zhang, Xiaofang Yan, Ying Sun, Liping Zhang, Jiangling Liu, Hao Dai, Libo Hou, Xi Li, Jing Li

**Affiliations:** 1 National Clinical Research Center for Cardiovascular Diseases, Fuwai Hospital, Chinese Academy of Medical Sciences and Peking Union Medical College, National Center for Cardiovascular Diseases, Beijing, China; 2 Fuwai Hospital Chinese Academy of Medical Sciences, Shenzhen, Shenzhen, People’s Republic of China; 3 Central China Sub-center of the National Center for Cardiovascular Diseases, Zhengzhou, People’s Republic of China; Stanford University School of Medicine and VA Palo Alto Health Care System, UNITED STATES OF AMERICA

## Abstract

**Aims:**

Heart failure (HF) is a serious cardiovascular condition resulting from abnormalities in multiple biological processes, affecting over 64 million people worldwide. We sought to expand our understanding of the genetic basis of HF and more specific NICM subtype in the East Asian populations and evaluate the biological pathways underlying subclinical left ventricular dysfunction.

**Methods and results:**

We conducted a meta-analysis of genome-wide association studies (GWAS) for all-cause HF in the East Asian populations (N cases ~ 13,385) and a more precise definition of nonischemic cardiomyopathy (NICM) subtype in multi-ancestry populations (N cases~3,603). We identified a low-frequency East-Asian enriched coding variant near *MYBPC3* and a NICM specific locus. Follow up analyses demonstrated male-specific HF association at the **M*YBPC3* locus, and highlighted *SVIL* as a candidate causal gene for NICM. Moreover, we demonstrated that *SVIL* deficiency aggravated cardiomyocyte hypertrophy, apoptosis and impaired cell viability in phenylephrine (PE)-treated H9C2 cells. In addition, the gene expression level of B-type natriuretic peptide (*BNP*) which was deemed as a hallmark for HF was further elevated by *SVIL* silencing in PE-stimulated H9C2 cells. RNA-sequencing analysis of H9C2 cells revealed that the function of *SVIL* might be mediated through pathways relevant to regulation and differentiation of heart muscle.

**Conclusion:**

These results enhance our understanding of the genetic architecture of HF in the East Asian populations, and provide important insight into the biological pathways underlying NICM and sex-specific relevance of the *MYBPC3* locus that warrants further replication in another datasets.

## Author summary

Heart failure (HF), a serious cardiovascular condition resulting from abnormalities in multiple biological processes, affects over 64 million people worldwide. Both genetic factors and exposure to environmental factors contribute to HF onset, with estimated heritability ranging from 26% to 34%. Genome-wide association studies (GWAS) have identified 106 susceptibility loci for HF, primarily in populations of European ancestry. However, the genetic architecture of HF in the East Asian populations remains to be explored. Therefore, we carried out a meta-analysis of genome-wide analysis for all-cause HF in the East Asian populations and more precise definition of nonischemic cardiomyopathy (NICM) subtype in multi-ancestry populations. We identified a low-frequency East-Asian enriched coding variant near the *MYBPC3* locus for HF and a NICM specific locus (*SVIL*). The effect of *MYBPC3* on risk of all-cause HF was more pronounced in males than in females. Furthermore, *SVIL* deficiency aggravated cardiomyocyte hypertrophy, apoptosis and impaired cell viability in phenylephrine (PE)-treated H9C2 cells. This work tackle the lack of population diversity in GWAS, and provide opportunities for further exploration of the biological mechanisms underlying the pathogenesis of HF and left ventricular remodeling.

## Introduction

Heart failure (HF), a serious cardiovascular condition resulting from abnormalities in multiple biological processes, affects over 64 million people worldwide [1] and has a prevalence of 1.3% among adults aged 35 and older in China [2]. HF imposes a significant economic burden, particularly in developed countries [3]. Despite advancements in diagnosis and treatment, mortality of HF remains high with 1-year mortality of more than 30% [4], and 5-year mortality of ~50% [5]. The wide spectrum of potential causes and the slow disease progression of HF make its prevention and treatment particularly challenging.

Both genetic factors and exposure to environmental factors contribute to HF onset, with estimated heritability ranging from 26% to 34% [6]. Genome-wide association studies (GWAS) have identified 106 susceptibility loci for HF, primarily in populations of European ancestry over the last decade [7–16]. However, many of these loci were associated with clinical HF risk factors, including coronary heart disease (CAD), atrial fibrillation (AF), and hypertension (HTN). Given the diverse etiologies and clinical heterogeneity of HF, there is a critical need to refine the HF phenotype and reveal the pathogenesis independent of these clinical HF risk factors.

Although recent GWASs of specific HF subtypes such as nonischemic cardiomyopathy (NICM) [8] and dilated cardiomyopathy (DCM), which is a primary cause of NICM [17,18], have been conducted in population of European ancestry, the genetic architecture of NICM in the East Asian populations remains to be explored. This is especially important given that the genetic associations previously identified in European ancestry may not be generalizable to other populations. Moreover, males and females with HF showed different characteristics, yet few GWAS have specifically examined sex differences in the genetic architecture of HF specifically. Thus, additional studies are needed to further elucidate the genetic architecture of HF independent of clinical HF risk factors and to determine the sex-specific relevance of HF-associated genetic variants in the East Asian populations.

In the present study, we sought to expand our understanding of the genetic basis of HF and more specific NICM subtype in the East Asian populations and evaluate the biological pathways underlying subclinical left ventricular dysfunction.

## Methods

### Ethics statement

The central ethics committee at Fuwai Hospital and local ethics committees at participating hospitals approved the China PEACE 5p-HF Study. The study was registered at www.clinicaltrials.gov (NCT02878811). The investigation conformed with the principles outlined in the Declaration of Helsinki. The central ethics committee at the China National Center for Cardiovascular Diseases approved this study (approval no. 2014–574). All enrolled participants provided written informed consent. All study participants provided informed consent, and the study was approved by the North West Multi-centre Research Ethics Committee.

### Study populations

The China Patient-centered Evaluative Assessment of Cardiac Events Prospective Heart Failure Study (China PEACE 5p-HF Study) enrolled patients hospitalized primarily for HF from 52 Chinese hospitals located in 20 provinces between August 2016 and May 2018. The protocol of the China PEACE 5p-HF Study has been published [19]. In brief, 4,866 patients aged 18 years or older, and hospitalized with a primary diagnosis of new-onset HF or decompensation of chronic HF assessed by local physicians, were enrolled in the study, and 4,363 were genotyped using Illumina genotyping arrays. In the current analysis, we used data from 3,972 all-cause HF cases and 1,787 NICM cases that passed genotyping QC procedures (S1 Table).

The China Health Evaluation And risk Reduction through nationwide Teamwork (ChinaHEART) project is a government funded public health programme designed for screening of cardiovascular disease risk and for intervention in community based populations throughout China. The design of ChinaHEART project has been described previously [20]. Briefly, from 20 November 2014–31 December 2021, 351 county level regions in all 31 provinces in the mainland of China were selected as study sites to provide diversity in geographical distribution, population structure, and exposure to risk factors and disease patterns. Study site selection also considered population size, population stability, and local capacity to support the project. A total of 4.5 million residents were recruited, and 14,853 were genotyped using Illumina genotyping arrays. In this study, a subset of 11,171 controls who were free from cardiovascular disease (CVD) and HF at baseline and during follow up between 2014 and 2017 were available as control subjects for the China PEACE 5p-HF Study (S1 Table).

Participating cohorts in the BioBank Japan (BBJ) have been previously described in detail [21]. Briefly, BBJ is a hospital-based Japanese national biobank project including data from approximately 200,000 patients enrolled between 2003–2007. Participants were recruited at 12 medical institutes throughout Japan.

The UK Biobank (UKB) recruited participants between 40–69 years of age who were registered with a general practitioner of the UK National Health Service (NHS). Between 2006–2010, a total 503,325 individuals were included. Detailed methods used by the UKB have been described elsewhere [22].

### Phenotyping

All-cause HF cases included participants with a clinical diagnosis of HF of any aetiology from the China PEACE 5p-HF Study. To avoid any misclassification of cases, we also excluded samples with a diagnosis of hypertrophic cardiomyopathy (HCM) due to the substantial Mendelian inheritance pattern of hypertrophic cardiomyopathy. Among all-cause HF patients, nonischemic cardiomyopathy (NICM) was defined on the absence of coronary heart disease (CHD), acute myocardial infarction (AMI) and ischemic cardiomyopathy. Non-HF controls were those who were free from cardiovascular disease (CVD) and HF at baseline and during follow up between 2014 and 2017 from the ChinaHEART project to eliminate the possibility of CVD individuals progressing to HF in controls. This definition strategy resulted in 3,972 all-cause HF cases, 1,787 NICM cases and 11,171 controls (S1 Table). **All-cause HF in UKB** was defined as (i) Self-reported history of HF or cardiomyopathy; or (ii) hospitalization for or death due to ICD-10 code for hypertensive heart disease, cardiomyopathy or HF (I11.0, I13.0, I13.2, I25.5, I42.0, I42.5, I42.8, I42.9, I50.0, I50.1, I50.9); or (iii) hospitalization due to ICD-9 code for HF or other primary cardiomyopathies (4254, 4280, 4281, 4289); **excluding** individuals with history of hypertrophic cardiomyopathy, or hospitalization for or death due to ICD-10 code for hypertrophic cardiomyopathy (I42.1, I42.2) (S1 Table). **NICM in UKB** was defined as (i) Hospitalization for or death due to ICD-10 code for dilated cardiomyopathy or left ventricular failure (I42.0, I50.1); **or** (ii) Hospitalization due to ICD-9 code for left heart failure (4281); **excluding** individuals with history of coronary artery, or history of hypertrophic cardiomyopathy, or hospitalization for or death due to ICD-10 code for hypertrophic cardiomyopathy (I42.1, I42.2). **HF in BBJ** was defined as patients with heart failure, diagnosed by physicians at the cooperating hospitals (S1 Table).

### Genotyping and imputation

Genome-wide genotyping of single-nucleotide polymorphisms (SNPs) in the China PEACE 5p-HF Study and the ChinaHEART project was performed on the Illumina Infinium Global Screening Array chip. Based on sample quality control procedures described in S2 Table, we excluded duplicate samples (positive controls) and samples with a low call rate (<90%), samples with first-degree relatives (prioritizing the cases and/or samples with higher call-rates from each pair) and samples with discordant ethnicity from Han population. Quality control procedures for genotyped SNPs excluded SNPs with significant departure from Hardy-Weinberg Equilibrium (HWE) in controls (P < 1 × 10^− 6^) and SNPs with a low call rate <97% (S3 Table). This resulted in 640,842 autosomal SNPs that were available for imputation in 15,143 participants.

Imputation procedures were performed using IMPUTE2 [23] and SHAPEIT2 [24] based on phase3 1000 G worldwide panel. Analyses with the imputed data set excluded SNPs with INFO scores <0.8 and with minor allele frequencies (MAF) <0.1% (S3 Table). This resulted in 6,976,847 autosomal SNPs that were available for a GWAS analysis in 15,143 Han Chinese subjects.

### GWAS for HF and NICM in the China PEACE 5p-HF/ChinaHEART

Autosomal SNPs were tested for association with HF and NICM using firth logistic regression, which applies firth correction to remove the bias of standard maximum likelihood estimates and retained good control of the type 1 error [25]. GWAS analyses for HF were also conducted in a sex-specific fashion. GWAS analyses were performed with regenie [25] which fit a whole-genome regression model for binary phenotypes with adjustment for age, sex (except for sex-stratified GWAS), and the first 10 principal components. In addition to the genomic control factor (λ), we also used LD Score regression to evaluate stratification in the China PEACE 5p-HF/ChinaHEART results. This approach can provide a more accurate correction factor than genomic control in GWAS with large sample sizes and help distinguish between inflation that is due to true genetic signals from inflation that is due to stratification [26]. The genome-wide significance threshold was set at P = 5.0 × 10^-8^. Manhattan and quantile-quantile plots were constructed using ‘qqman’ package (v0.1.8) in R. All power calculations were carried out using QUANTO [27].

### Meta-analyses for HF in the China PEACE 5p-HF/ChinaHEART and BBJ

Publicly available summary statistics for HF with 8,678,732 SNPs from the BBJ with 9,413 cases and 203,040 controls were downloaded [11]. We carried out a fixed-effects meta-analysis in 13,385 cases and 214,211 controls with 5,887,003 SNPs common to the China PEACE 5p-HF/ChinaHEART and the BBJ datasets assuming an additive model, as implemented in METAL [28]. Similar to the GWAS analysis in the PEACE 5p-HF/ChinaHEART alone, the genomic control factor (λ) and LD Score intercept were used to evaluate stratification in the meta-analysis results. The genome-wide threshold for significant association was set at P = 5.0 × 10^-8^. A locus was defined as novel if our lead SNP was > 1Mb away or in weak or no (r ^2 ^≤ 0.1) linkage disequilibrium (LD) with the lead variants at the 106 previously reported loci for HF [7–16]. We controlled the potential bias of population structure by performing the heterogeneity test, and the threshold for significant heterogeneity was set at P < 0.1. Replication of the 106 known HF loci was considered significant at a Bonferroni-corrected threshold of P = 4.7 × 10^-4^ (0.05/106).

### Sex-stratified Meta-analyses for HF in the China PEACE 5p-HF/ChinaHEART and BBJ

Sex-stratified meta-analyses for all-cause HF association was carried out with GWAS result in the China PEACE 5p-HF/ChinaHEART and summary level data in BBJ [11] in males (8,419 HF cases and 107,613 controls) and females (4,966 HF cases and 106,598 controls) separately (S1 Table). Cochran’s Q statistics for heterogeneity P-value between males and females (P-het) was considered significant at the P < 0.05 for testing the *MYBPC3* locus.

### Meta-analyses for NICM in the China PEACE 5p-HF/ChinaHEART and the UKB

Publicly available summary statistics for NICM with 7,736,183 SNPs from the UKB with 1,816 cases and 388,326 controls were downloaded [8]. We carried out a fixed-effects meta-analysis in 3,603 cases and 399,497 controls with 4,567,081 SNPs common to the China PEACE 5p-HF/ChinaHEART and the UKB datasets assuming an additive model, as implemented in METAL [28]. The genome-wide threshold for significant association was set at P = 5.0 × 10^-8^. A locus was defined as novel if our lead SNP was > 1Mb away or in weak or no (r ^2^ ≤ 0.1) linkage disequilibrium (LD) with the lead variants at the 106 previously reported loci for HF [7–16]. We controlled the potential bias of population structure by performing the heterogeneity test, and the threshold for significant heterogeneity was set at P < 0.1.

### Association of identified loci with HF risk factors

The lead variants at the newly identified HF loci were evaluated for association with various HF risk factors from previously published studies, including body mass index (BMI) [29], blood pressure (systolic blood pressure, diastolic blood pressure, and pulse pressure) [30], plasma lipids (total cholesterol, low-density lipoprotein cholesterol, high-density lipoprotein cholesterol, and triglycerides) [31], estimated glomerular filtration rate (eGFR) [32], atrial fibrillation (AF) [33], coronary artery disease (CAD) [34], myocardial infarction (MI) [35], and type 2 diabetes (T2D) [36]. A Bonferroni-corrected P = 9.6 × 10^-4^ (0.05/52) for testing 4 loci and 13 traits were considered as the threshold for significant associations with HF risk factors.

### Association of identified loci with cardiac magnetic resonance imaging (MRI)-derived left ventricular measure

Evaluation of the identified HF loci for association with cardiac magnetic resonance imaging (MRI)-derived left ventricular measure, including left ventricular ejection fraction (LVEF), left ventricular end-diastolic volume (LVEDV), left ventricular end-systolic volume (LVESV), and stroke volume (SV) were carried out using a previously published study in the UKB [37]. Associations with P≤ Bonferroni-corrected threshold for testing 4 SNPs and 4 traits (0.05/16 = 3.1 × 10^-3^) were considered significant.

### Comparison of identified loci across ancestries

To understand the genetic basis of HF and NICM in the East Asian population, we also compared the difference of the identified loci (*SVIL* and *MYBPC3*) between allele frequencies and effect sizes in the current study and present studies on DCM [17,38,39], HCM [40], and cardiac MRI-derived traits [37,41,42] across different populations.

### Data-driven expression-prioritized integration for complex traits (DEPICT)

Additional gene prioritization analysis and tissue enrichment analysis were carried out by DEPICT (version 1.1) [43] using 207 SNPs with false discovery rates (FDRs) < 0.05 (P < 1.75x10^-6^) associated with HF that were identified in the meta-analysis with the China PEACE 5p-HF/ChinaHEART and BBJ. Both nominal P-values and FDRs were calculated for gene set enrichment and tissue enrichment.

### Expression quantitative trait locus (eQTL) and splice quantitative trait locus (sQTL) analyses

Functional evaluation of SNPs at the 4 HF loci and prioritization of candidate causal genes was determined using multi-tissue expression (eQTL) and splice (sQTL) data from the GTEx Project (version 8) [44] and other datasets, such as the eQTLgen Consortium [45], the Human Protein Atlas (HPA) [46] or previously published studies available through the PhenoScanner database [47]. Consideration was only given to *cis* eQTLs and *cis* sQTLs with P < 5.0x10^− 5^ that were derived from our lead GWAS variants.

### Sensitivity analyses

To harmonize phenotypes between China PEACE 5p-HF cohort and the UKB, we further did sensitivity analyses for the NICM phenotype in the China PEACE 5p-HF cohort by 1) keeping samples (N case = 789) with either doctor diagnosed dilated cardiomyopathy or LVEF ≤ 40% in the echocardiographic data as we did not have information for doctor diagnosed left ventricular failure; 2) dividing NICM phenotype into heart failure with reduced ejection fraction (HFrEF) (N case = 770) and heart failure with preserved ejection fraction (HFpEF) (N case = 934).

### Genetic correlations

We used LDSC [26] (v.1.0.1) to estimate the genetic correlations (rg) between HF/NICM and 10 relevant risk factors (BMI [48], HTN [49], AF [33], CAD [50], T2D [50], heart rate (HR) [49], chronic kidney disease (CKD) [51], MI [35], LVEF [37] and HCM [40]). We computed our own linkage disequilibrium (LD) score using the China PEACE 5p-HF/ChinaHEART dataset, which was used as the reference panel. Hypothesis tests were performed using a null hypothesis of 0, using two-sided tests.

### Conditional GWAS analyses

Conditional GWAS analyses were performed using a multitrait-based conditional and joint analysis (mtCOJO) method [52] implemented in GCTA (v.1.94.4), which we used to estimate the genetic effects of disease conditioning on BMI, HTN, AF, CAD, T2D, HR, CKD, MI, LVEF, and HCM. To perform the analysis, we used summary statistics from GWAS of BMI in 523,818 subjects [48], HTN in 144,793 cases and 313,761 controls [49], AF in 65,446 cases and 522,744 controls [33], CAD in 154,194 cases, and 278,454 controls [50], T2D in 154,194 cases, and 278,454 controls [50], HR in 458,969 subjects [49], CKD in 64,164 cases, and 561,055 controls [51], MI in ~61,000 cases and ~577,000 controls [35], LVEF in ~36,000 subjects [37], and HCM in 5,900 cases and 68,359 controls [40]. We used the LD scores computed from the China PEACE 5p-HF/ChinaHEART dataset.

### Cell culture and treatment

H9C2 cells obtained from ATCC (Manassas, VA, USA) were cultured in Dulbecco’s modified Eagle’s medium (iCell Bioscience) supplemented with 10% fetal bovine serum (Gibco) and 1% penicillin/streptomycin (Gibco) in a humidified incubator with 5% CO_2_ at 37°C. When the cell coverage rate reached 70%, trypsin (Gibco) was used to dissociate H9C2. In the cell model of (murine) cardiomyocyte hypertrophy, phenylephrine (PE) was utilized as agonists according to previous studies [53–55]. Firstly, H9C2 cells were seeded in 12-well culture plates or confocal dishes at the density of 2 × 10^5^ cells per well, and then incubated in complete medium for 24 h. Next, serum-free medium was adopted for another 24 h, siRNA transfection could be performed in the meanwhile. Subsequently, PE dissolved in PBS was added to the medium at a final concentration of 100 μM for 48 h. Equal volumes of PBS (vehicle) were administered in control group. After incubation, subsequent experiments were performed, including total RNA extraction for RT-qPCR and cell surface area measured by staining of FITC-labelled Phalloidin. All the cell experiments were repeated three times independently.

### SiRNA transfection

The expression levels of *SVIL* in H9C2 cells were suppressed by siRNA-mediated knockdown targeting *SVIL* gene. Lipofectamine RNAimax (Invitrogen) was utilized for siSVIL (RiboBio) or non-targeting siRNA (siCtrl) transfection following the manufacturer’s instruments.

### *In vitro* hypertrophy assays

H9C2 cells prepared for immunofluorescence microscopy were seeded in confocal dishes and exposed to hypertrophic agonist PE for 48 h. After activation, cells were fixed in 4% paraformaldehyde and stained with FITC-conjugated phalloidin (Biosharp) for 30 min. DAPI was used for cell nuclei staining. A Leica TCS SP8 STED microscope and Image J software was used to acquire images and measure cell surface area. Fold changes in the average surface area of treated cells were calculated relative to that of untreated ones.

### Quantitative real-time PCR

Total RNA was extracted from H9C2 cells using the TRIzol reagent (Invitrogen) as described in the manufacturer’s manual. The obtained RNA was then used for cDNA synthesis with HiScript III RT SuperMix (Vazyme) and subsequently performed qPCR with AceQ qPCR SYBR Green Master Mix (Vazyme). The mRNA expression levels of *BNP* and *ANP* were normalized to the housekeeping gene *GAPDH*. Relative gene expression levels were calculated using ΔΔCt-method. The following primers were used for qRT-PCR: BNP, forward, 5’-GAACAATCCACGATGCAGAAGC-3’ and reverse, 5’-GGGCCTTGGTCCTTTGAGAG-3’; ANP, forward, 5’-GAGGAGAAGATGCCGGTAG-3’ and reverse, 5’- CTAGAGAGGGAGCTAAGTG-3’.

### Western blot

For immunoblotting, cells were homogenized in cell lysis buffer (150 mM NaCl, 50 mM HEPES pH 7.4, 1% Triton X-100). Bicinchoninic Acid (BCA) Protein Assay Kit (Thermo Fisher) was used to determine the protein concentration of the samples. Samples were loaded on 10% SDS-PAGE gels and then transferred to polyvinylidene fluoride (PVDF) membranes. The PVDF membrane was incubated in blocking buffer (Beyotime) for 1 h at room temperature. Subsequently, primary antibodies diluted as 1:1000 were applied to the blot overnight at 4°C with gentle rocking. Antibodies were purchased as follows: supervillin (Santa; sc-53556), caveolin-3 (Proteintech; 28358–1-AP), α-sarcoglycan (Santa; ab189254), GAPDH (ZSGB-bio; TA-08). HRP‑labeled antibody diluted as 1:5000 was used as the secondary antibody and incubated with the membrane for 1 h. Enhanced chemiluminescence (ECL) solution was added on the membrane for its development and photograph.

### Cell apoptosis detection

Annexin V-FITC Apoptosis Detection Kit (abcam, ab14085) was used to evaluate the effect of *SVIL* deficiency and PE treatment on H9C2 cell apoptosis. In brief, H9C2 cells were collected and washed twice with PBS precooled at 4°C. After centrifugation, the supernatant was discarded, and the cell pellet was resuspended with 500 μL of 1 × binding buffer. Then, 5 μL annexin V-FITC and 5 μL propidium iodide (PI) were added, mixed gently, and incubated at room temperature in darkness for 15 min. A cell group without staining was set as negative control, and a single dye staining group (stained with only one dye) were set for the voltage of flow cytometry and its compensation, and the apoptosis rate of the cells was detected. Finally, the data were analyzed and plotted with FlowJo V10.

### Cell viability assay

The Cell Counting Kit 8 (CCK8) (YEASEN) was applied to measure cell viability. H9C2 cells were seeded into 96-well plates at a density of 2 × 10^3^ cells/well. After 48 h-treatment, 10 μL CCK8 solution was added into the wells and the cells were incubated for 4 h at 37°C in the dark. Finally, the absorbance was measured at 450 nm by a microplate reader.

### Library construction for RNA-seq and sequencing procedures

Total RNA was isolated using MJzol animal RNA Extraction Kit (MagBeads). Paired-end libraries were synthesized using the TruSeq RNA Sample Preparation Kit (Illumina, USA) following TruSeq RNA Sample Preparation Guide. Briefly, the poly-A containing mRNA molecules were purified using poly-T oligo-attached magnetic beads. Following purification, the mRNA was fragmented into small pieces using divalent cations under 94°C for 8 min. The cleaved RNA fragments were copied into first strand cDNA using reverse transcriptase and random primers, followed by second strand cDNA synthesis using DNA Polymerase I and RNase H. These cDNA fragments then went through an end repair process, the addition of a single ‘A’ base, and then ligation of the adapters. The products were purified and enriched with PCR to create the final cDNA library. Purified libraries were quantified by Qubit 2.0 Fluorometer (Life Technologies, USA) and validated by Agilent 2100 bioanalyzer (Agilent Technologies, USA) to confirm the insert size and calculate the mole concentration. Cluster was generated by cBot with the library diluted to 10 pM and then sequenced on the Illumina Nova 6000 (Illumina, USA).

### Data analysis and identification of differentially expressed genes (DEGs)

Sequencing raw reads were preprocessed by filtering out rRNA reads, sequencing adapters, short-fragment reads and other low-quality reads. We used Hisat2 (version:2.0.4) [56] to map the cleaned reads to the rat Rnor_6.0 reference genome with two mismatches. After genome mapping, Stringtie (version:1.3.0) [57,58] was used with a reference annotation to generate fragments per kilobase per million reads (FPKM) values for known gene models. Differentially expressed genes (DEGs) were identified using edgeR [59]. The p-value significance threshold in multiple tests was set by the FDR. The fold-changes were also estimated according to the FPKM in each sample. The DEGs were selected using the following filter criteria: FDR ≤ 0.05 and fold-change ≥2.

### Gene ontology (GO) enrichment analysis

For functional enrichment analysis, all DEGs were mapped to terms in the GO databases, and then significantly enriched GO terms were defined with P < 0.05 and rich factor ≥5 as the threshold. GO term analysis was classified into three subgroups, namely biological process (BP), cellular component (CC) and molecular function (MF).

## Results

### Identification of 3 loci for all-cause HF among East-Asian populations

**Fig 1:**
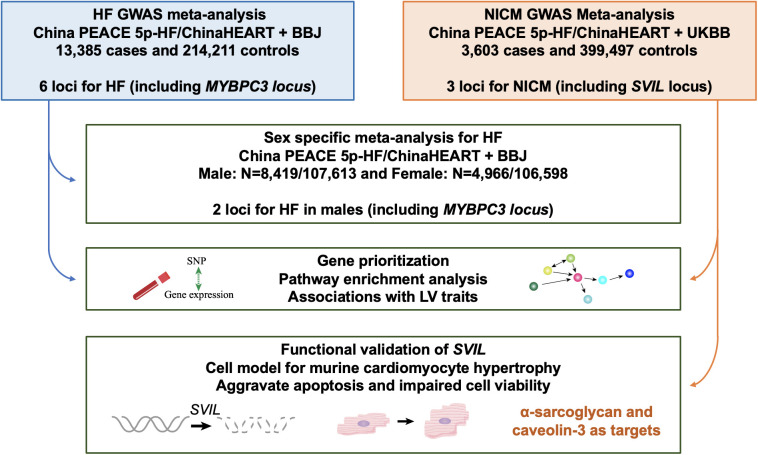
Overview of genetic and functional analyses. A GWAS was first carried out in 3,972 all-cause HF cases and 11,171 controls in the China PEACE 5p-HF/ChinaHEART.We then performed a fixed-effects meta-analysis with BBJ in 13,385 cases and 214,211 controls. In parallel, we carried out a GWAS for HF subtype in 1,787 NICM cases and 11,171 controls in the China PEACE 5p-HF/ChinaHEART, followed by a meta-analysis with the UKB in 3,603 cases and 399,497 controls. Sex-specific meta-analysis for HF identified a variant at the *MYBPC3* locus to be male-specific. Gene prioritization and pathway enrichment analysis were carried out. Functional validation of *SVIL* was conducted in cell model for murine cardiomyocyte hypertrophy. *SVIL* deficiency aggravated cardiomyocyte hypertrophy, apoptosis and impaired cell viability in phenylephrine (PE)-treated H9C2 cells.

**Fig 2:**
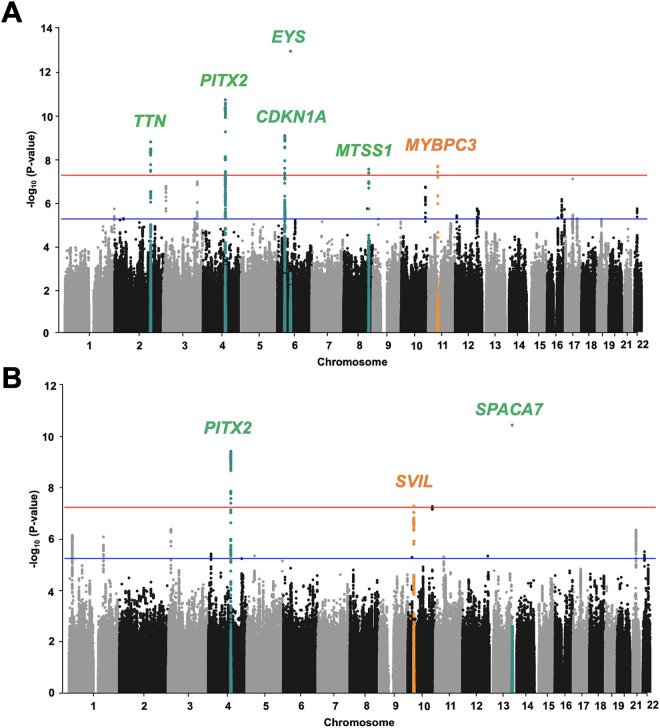
Manhattan plot of results from GWAS meta-analysis for HF and NICM. (A) Of the 6 genome-wide significant loci in the meta-analysis for HF, 2 (*PITX2* and *CDKN1A*) were previously reported in HF GWAS, and 2 (*TTN* and *MTSS1*) were reported in the multi-trait GWAS of HF and cardiac MRI traits GWAS, whereas the other locus (*EYS*) was a singleton (dark green dots). One locus (*MYBPC3*) was previously not identified in the HF GWAS in the East Asian populations (orange dots). The fixed-effects meta-analysis included a total of 13,385 HF cases and 214,211 controls from the China PEACE 5p-HF/ChinaHEART (3,972 HF cases and 11,171 controls) and the BBJ datasets (9,413 HF cases and 203,040 controls) with 5,887,003 SNPs common to both datasets. (B) Of the 3 genome-wide significant loci in the meta-analysis for NICM, 1 locus (*PITX2*) was previously reported, whereas the other locus (*SPACA7*) was a singleton (dark green dots). The other one (*SVIL*) was previously not identified in the NICM meta-analysis between the East Asian population and Europeans (orange dots). The fixed-effects meta-analysis included a total of 3,603 NICM cases and 399,497 controls from the China PEACE 5p-HF/ChinaHEART (1,787 NICM cases and 11,171 controls) and the UKB (1,816 NICM cases and 388,326 controls) with 4,567,081 SNPs common to both datasets. Genome-wide thresholds for significant (P = 5.0x10^-8^) and suggestive (P = 5.0x10^-6^) association are indicated by the horizontal red and dark blue lines, respectively.

To further elucidate the genetic architecture of heart failure, we first carried out a genome-wide association study (GWAS) in 3,972 all-cause HF cases and 11,171 controls in the China PEACE 5p-HF/ChinaHEART (S1 Table). Among them, 1,543 were classified as HFrEF (LVEF < 50%). CAD and MI were reported for 53.1% and 20.9% of the HF cases (S4 Table). However, this GWAS analysis failed to identify any genome-wide significantly HF-associated single-nucleotide polymorphisms (SNPs) with a minor allele frequency (MAF) > 1%, probably due to limited statistical power (S1 Fig). We next combined our GWAS results from the China PEACE 5p-HF/ChinaHEART with summary statistics for congestive heart failure from BBJ [11] in a fixed-effects meta-analysis that included a total of 13,385 HF cases and 214,211 controls with 5,887,003 SNPs common to both datasets (Fig 1 and S1 Table). This analysis revealed 88 significantly associated variants distributed across 6 loci (Figs 2A and S2 and S5 Table). Of these 6 loci, one locus, *MYBPC3*, was associated with HF at the genome significant level in the East Asian populations for the first time (Table 1 and Figs 2A and S3). Two loci, *TTN* and *MTSS*1**, had been previously reported in multi-trait GWAS of HF and cardiac MRI traits GWAS [12], while another locus, *EYS*, was a singleton, strongly suggesting a false-positive signal. The other two genome-wide significant loci, *PITX2* and *CDKN1A*, overlapped with the 106 previously known regions identified for HF [7–16] in populations of predominantly European ancestry (S6 Table). However, despite the P-values not exceeding the genome-wide significance level of 5.0 × 10^-8^, our meta-analysis did yield evidence for association of 47 of the 106 known HF loci at a Bonferroni-corrected threshold for testing 106 regions (P = 0.05/106 = 4.7 × 10^-4^), and all other 59 loci achieved a significance level of *P* < 0.05 (S6 Table). Thus, our meta-analyses in the East Asian populations, including data from the China PEACE 5p-HF/ChinaHEART and BBJ, successfully replicated association signals at all 106 previously known regions.

**Table 1:** 2 loci for heart failure (HF) and non-ischemic cardiomyopathy (NICM) in the meta-analysis.

SNP	CHR	BP (hg19)	Nearest Gene(s)	EA/NEA	EAF*	OR (95%CI)	P	P-het
				Meta-analysis for HF (13,385/214,211)**				
rs139616510	11p11.2	47,266,755	*MYBPC3*	T/C	0.005	1.73 (1.43-2.10)	1.9E-08	0.19
				Meta-analysis for NICM (3,603/399,497)***				
rs2182400	10p11.23	29,707,551	*SVIL*	G/A	0.53	1.16 (1.10-1.22)	4.5E-08	0.53

HF, heart failure; NICM, non-ischemic cardiomyopathy; CHR, chromosome; BP, base pair position (hg19); EA, effect allele; NEA, non-effect allele; EAF, effect allele frequency (*only available from the China PEACE 5p-HF/ChinaHEART); OR, odds ratio; 95%CI, 95% confidence interval; P-het, p-value for heterogeneity between the China PEACE 5p-HF/ChinaHEART and Biobank Japan (BBJ) for meta-analysis of HF, and between the China PEACE 5p-HF/ChinaHEART and the UK Biobank (UKB) for meta-analysis of NICM.

Number in parentheses indicate number of cases/controls.

**1 locus identified for HF in meta-analysis of the China PEACE 5p-HF/ChinaHEART and BBJ.

***1 locus identified for NICM in meta-analysis of the China PEACE 5p-HF/ChinaHEART and the UKB.

### Sex-stratified meta-analysis for HF in the China PEACE 5p-HF/ChinaHEART and BBJ

**Fig 3:**
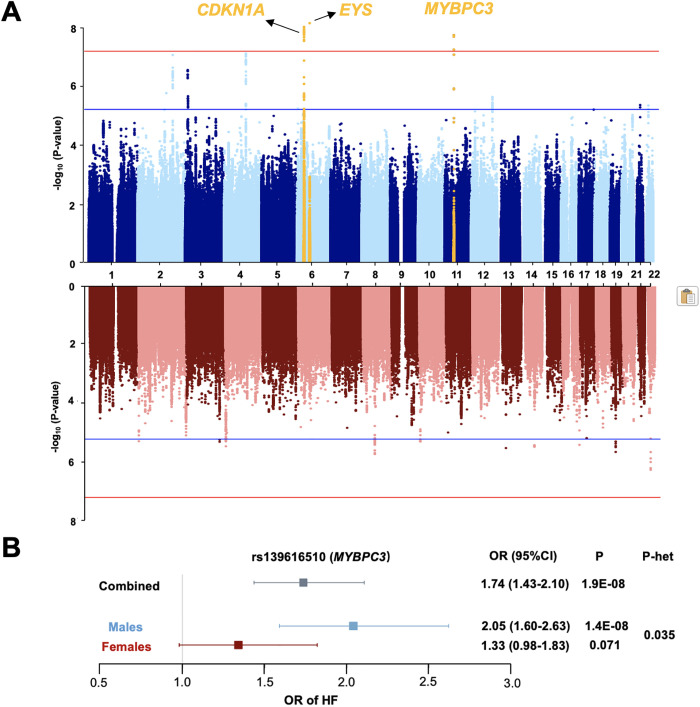
Sex-stratified GWAS meta-analysis for HF with the China PEACE 5p-HF/ChinaHEART and BBJ. (A) The Manhattan plot in males (8,419 HF cases and 107,613 controls) revealed 22 significantly associated variants distributed across 3 loci (*CDKN1A*, *EYS* and *MYBPC3* in yellow dots), whereas the Manhattan plot in females (4,966 HF cases and 106,598 controls) shows no genome-wide significantly associated SNPs with MAF > 1%. Genome-wide thresholds for significant (P = 5.0x10^-8^) and suggestive (P = 5.0x10^-6^) association are indicated by the horizontal red and dark blue lines, respectively. P-values are truncated at -log_10_(P)=8. (B) *MYBPC3* on 11p11.2 yielded nominal P-value for interaction with sex (P-het = 0.035), and the effect sizes were stronger in males (OR=2.05, 95% CI 1.60-2.63) than in females (OR=1.33, 95% CI 0.98-1.83).

We next carried out sex-stratified meta-analysis for all-cause HF with GWAS results from the China PEACE 5p-HF/ChinaHEART and summary level GWAS data from BBJ in males and females separately. The meta-analysis in males revealed 22 significantly associated variants distributed across three loci (Figs 3A and S4 and S7 Table). Of these, one locus (*MYBPC3*) was also associated with all-cause HF in the combined analysis (Table 1). Among the other two loci, one (*CDKN1A*) was a previously known HF-associated locus (S6 Table), while the *EYS* locus was a singleton, strongly suggesting a false-positive signal. Nonetheless, no genome-wide significant SNPs were identified in females (Figs 3A and S5). To further investigate sex differences, we compared association signals at the six HF susceptibility loci between males and females using the sex-stratified meta-analysis results. Interestingly, *MYBPC3* at 11p11.2 yielded a nominal P-value for interaction with sex (P-het < 0.05), with a stronger effect size in males (OR = 2.05, 95% CI 1.60-2.63, P = 1.4 × 10^-8^) than in females (OR = 1.33, 95% CI 0.98-1.83, P = 0.071) (Fig 3B and S8 Table).

### Identification of two loci for NICM

To facilitate the discovery of genetic signals that act independent of traditional HF risk factors, we performed a GWAS in 1,787 NICM cases and 11,171 controls from the China PEACE 5p-HF/ChinaHEART (Fig 1 and S1 Table). Among them, 770 were classified as HFrEF. Hypertension and type 2 diabetes were reported for 49.1% and 18.9% of the NICM cases (S4 Table). The NICM GWAS was then meta-analyzed with the UKB [8], since the NICM GWAS result is not available from BBJ. This meta-analysis for NICM in 3,603 cases and 399,497 controls resulted in 52 significantly associated variants distributed across three loci (S9 Table and Figs 2B and S6). Of these loci, one (*SVIL*) was associated with NICM at the genome significant level in the meta-analysis between East Asian population and Europeans for the first time herein (Table 1 and S7 Fig). Among the other two loci, one (*SPACA7*) was a singleton, which strongly suggests a false-positive signal, whereas the other locus (*PITX2*) overlapped with one of the 106 previously known regions identified for HF [7–16] (S6 Table).

### Association of identified loci with HF risk factors and MRI traits

We next explored whether the identified loci were associated with HF risk factors and MRI traits. Based on publicly available data, the risk allele of lead variants at the *TTN* and *MTSS1* loci identified in the meta-analysis for all-cause HF were associated with increased risk of atrial fibrillation (AF) [33] at a Bonferroni-corrected significance level of 9.6x10^-4^ (0.05/52) for testing four loci and thirteen traits (S10 Table). Moreover, the *TTN*, *MTSS1*, and *SVIL* loci were associated with reduced left ventricular ejection fraction (LVEF) and increased left ventricular end-systolic volume (LVESV) in a recent large-scale GWAS of cardiac MRI-derived LV traits [37] (S11 Table). Since the lead SNP rs139616510 at the *MYBPC3* locus has a low MAF (~ 0.006%) in the UKB and a relatively higher MAF (0.5%) in East Asians, we alternatively evaluated its association with echocardiographic traits in a subset of the China PEACE 5p-HF Study who went through echocardiography (N ≈ 3000). Consistent with its role in left ventricular remodeling, the *MYBPC3* locus was associated with decreased LVEF and increased left ventricular end-diastolic volume (LVEDV) (S11 Table).

Comparisons of the differences between allele frequencies and effect sizes of the identified loci across different populations have been made. As shown in S12 Table, the G allele of rs2182400 at *SVIL* was associated with increased risk of NICM (EAF = 0.53, OR=1.14, P = 6.4 × 10^-4^) in the China study, and with similar effects in Europeans (EAF = 0.24, OR=1.10, P = 1.6 × 10^-9^). In the HCM GWAS, the same variant had a protective effect on HCM_MTAG (OR=0.87, P = 1.1 × 10^-8^) and HCM _SARC-_ (OR=0.86, P = 2.7 × 10^-6^) in Europeans. The other variant (rs10740811) at *SVIL* was associated with increased descending thoracic aorta (EAF = 0.41, beta = 0.06, P = 6.4 × 10^-25^), but this variant is not significantly associated with NICM in the China study (EAF = 0.45, P = 0.303). As for the *MYBPC3* locus, the low-frequency East-Asian enriched coding variant rs139616510 only yields a MAF of ~0.006% in the UKB, the other European enriched rare variants (rs182427065, rs79098768 and rs117879887) associated with HCM and its subtypes [40] only have a MAF of 0 in the China study. The common variant rs11570078 at *MYBPC3* was associated with DCM_MTAG (EAF = 0.87, OR=0.92, P = 1.6 × 10^-8^), but this variant is not significantly associated with HF in the China study (EAF = 0.95, P = 0.262) (S13 Table).

### Enrichment of HF-associated variants in biological pathways or tissues

To gain biological insight into the newly identified HF loci, we used bioinformatics tool to carry out enrichment analyses for regulatory elements and pathways. A Data-driven Expression-Prioritized Integration for Complex Trait (DEPICT) analysis revealed that the most significantly enriched biological pathways and tissues were abnormal myocardial trabeculae morphology, thin ventricular wall, actin monomer binding, abnormal heart development, heart atria, and atrial appendage (S14 and S15 Tables). Taken together, these results further highlight the myocardial structure and heart development as an important component in the pathophysiology of HF.

### Sensitivity analysis

The lead SNP rs2182400 at *SVIL* was significantly associated with NICM in the sensitivity analysis by keeping samples (N case = 789) with either doctor diagnosed dilated cardiomyopathy or LVEF ≤ 40% in the echocardiographic data (OR=1.20, 95%CI 1.08-1.33, P = 7.5 × 10^-4^), whose effect is stronger than the original NICM analysis (N case = 1,787, OR=1.14, 95%CI 1.06-1.23, P = 6.4 × 10^-4^). Out of the 1,787 NICM cases in the China PEACE 5p-HF cohort, 83 samples did not have echocardiographic data. Therefore, we conducted sensitivity analyses for the NICM phenotype in HFrEF (N case = 770) and HFpEF (N case = 934) in the China PEACE 5p-HF cohort. The lead SNP rs2182400 at *SVIL* was more significantly associated with NICM in HFrEF (OR=1.20, 95%CI 1.08-1.34, P = 6.6 × 10^-4^) compared to HFpEF (OR=1.12, 95%CI 1.01-1.24, P = 0.027) and the original NICM analysis (OR=1.14, 95%CI 1.06-1.23, P = 6.4 × 10^-4^) (S16 Table).

### Genetic correlations

Positive genetic correlations were observed across between HF and various risk factors except for LVEF (rg = -0.271, P = 0.123), with estimates ranging from 0.242 (*P* = 0.241) between HR and HF to 0.585 (*P* = 0.042) between T2D and HF (S17 Table). As we excluded HCM from HF definition, there was limited genetic correlation between HCM and HF (rg = 0.069, P = 0.772). As expected, the genetic correlations were weak between CAD/MI and NICM (rg = 0.179, P = 0.211 for CAD, and rg = 0.213, P = 0.232 for MI).

### Conditional GWAS analyses

To estimate the extent to which the NICM risk effects of *SVIL* were related to relevant risk factors (BMI, HTN, AF, CAD, T2D, HR, CKD, MI, LVEF and HCM), we conditioned the NICM GWAS summary statistics on these risk factors using mtCOJO. Conditioning on LVEF and HCM partially attenuated the association signal at the *SVIL* locus, implying genetic effects of *SVIL* on NICM risk may overlap with known genetic influences on LVEF (OR_conditioned = 1.12, 95%CI 1.06-1.18, P_conditioned = 1.3 × 10^-4^) and HCM (OR_conditioned = 1.12, 95%CI 1.06-1.18, P_conditioned = 3.4 × 10^-5^). Genetic association estimates were robust to conditional analysis on other risk factors (BMI, HTN, AF, CAD, T2D, HR, CKD, and MI), suggesting that the effects of *SVIL* on NICM were independent of these factors (S18 Table).

### Prioritization of positional candidate genes

**Fig 4:**
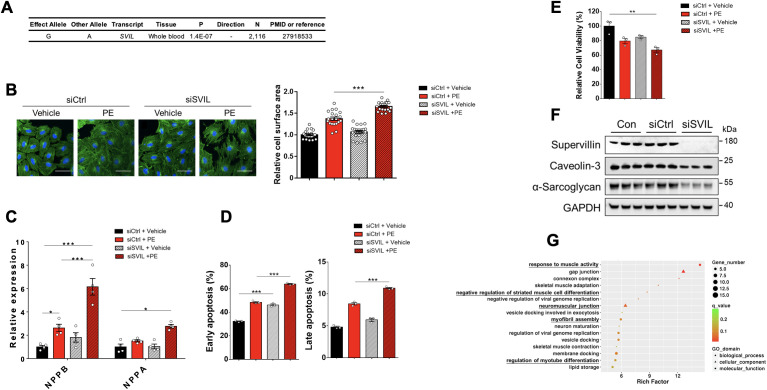
*SVIL* inhibition exacerbates hallmark features of pathologic (murine) cardiomyocyte remodeling. (A) The association of NICM risk allele (G) with lower expression level of *SVIL* in whole blood. (B) Left panel: The size and cytoskeletal morphology of cardiomyocyte H9C2 visualized by FITC-labelled Phalloidin were used to evaluate the effect of *SVIL* deficiency on the PE-induced cardiomyocyte hypertrophy. Cell nuclei were stained by DAPI. Scale bar = 50 μm. Right panel: Cell size was measured and analyzed by Image J software. N = 20. (C) RT-qPCR revealed altered gene expressions of biomarkers in heart failure (the mRNA of *BNP* and *ANP*) affected by *SVIL* deficiency on the PE-treated cells. N = 4. (D) The percentages of early (left) and late (right) apoptosis cells were displayed as histograms. H9C2 cells treated by *SVIL* siRNA interference or/and PE were staining with Annexin V-FITC/PI and detected signals through flow cytometry. N = 3. (E) Cell viability assay by CCK8. N = 3. (F) Western blot analysis of the expression levels of supervillin, caveolin-3 and α-sarcoglycan induced by *SVIL* silencing. GAPDH was used as a loading control. N = 3. (G) DEG analysis and functional enrichment analysis based on *SVIL-*silencing cardiomyocyte compared to that in control. GO terms assigned to biological process, cellular component, and molecular functions. Abbreviations: PE, Phenylephrine. Data are shown as mean ± SEM. *P < 0.05, **P < 0.005; ***P < 0.001.

To identify candidate causal genes at the newly identified loci, we first used multi-tissue gene expression (eQTL) and splice (sQTL) data from the GTEx Project (version 8) [44] and other datasets, such as the eQTLgen Consortium, or previously published studies available through the PhenoScanner database [47]. For loci on 2q31.2, 8q24.13 and 10q11.23, at least one candidate gene could be prioritized based on the lead SNP yielding a *cis* eQTL in one or more tissues relevant to HF, such as aorta, heart, and blood (S19 Table). Positional candidate genes with highly significant *cis* eQTLs were *TTN*, *TTN-AS1* and *FKBP7* on 2q31.2, *MTSS1* on 8q24.13, and *SVIL*, *SVIL-AS1*, and *PTCHD3P1* on 10q11.23 (Sl9 Table). We further prioritized loci by focusing on positional candidates that yielded eQTLs in heart tissues (atrial appendage, left ventricle) and skeleton muscle, since the heart is composed mostly of muscle and stromal tissue. *SVIL* on chromosome 10q11.23 yielded significant eQTLs in these HF-related tissues (S19 Table). In support of the gene regulatory effect, the lead SNP on chromosomes 10q11.23 also displayed significant *cis* sQTL with *SVIL* in the GTEx Project (S20 Table). We have also assessed the chromatin interaction data from ENCODE, and found rs2182400 is associated with enhancer histone marks in heart, left ventricle, and skeletal muscle. Supervillin, encoded by the *SVIL* gene, is a actin-binding protein [60] crucial for muscle fiber integrity [61] and *SVIL* has the highest expression in skeletal and heart muscle in the HPA and GTEx Project. In addition, the lead SNP on chromosome 10q11.23 yielded *cis* eQTLs for *SVIL* in whole blood, where the NICM risk allele (G) was associated with decreased expression of *SVIL* (Fig 4A and S19 Table). Taken together, we prioritized *SVIL* as a strong causal positional candidate gene for functional validation (Fig 1).

### Reduction of *SVIL* aggravates the cardiomyocyte injury in the cell model of (murine) cardiomyocyte hypertrophy

In order to assess the role of *SVIL*, H9C2 cells were transfected with siRNA targeting *SVIL*, and the knockdown efficiency was measured by quantitative real-time PCR (RT-qPCR) (S8 Fig). In the cell model of (murine) cardiomyocyte hypertrophy, H9C2 cells were exposed to phenylephrine (PE) for 48 hours based on previous studies [62–64]. Visible hypertrophic response was observed in PE-treated cells shown as cytoskeletal rearrangement in F-actin stress fibers (stained by FITC-labelled Phalloidin). This hypertrophy was more pronounced in *SVIL*-silenced cells (Fig 4B**, left panel**), where the cell surface area increased by 1.2-fold compared to the PE-only group (*P* < 0.001, Fig 4B**, right panel**). Overall, the data of cell morphology indicated that *SVIL* deficiency was prone to cause cardiomyocyte hypertrophy. Then, RT-qPCR was carried out to detect the gene expression of B-type natriuretic peptide (*BNP*) and atrial natriuretic peptide (*ANP*), whose elevation were used to diagnose heart failure. As shown in Fig 4C, both expressions of *BNP* and *ANP* were significantly increased in siSVIL+PE group in contrast to those in control (siCtrl+Vehicle), although *SVIL* silencing alone had no obvious effect on those gene expressions. Notably, *SVIL* deficiency further raised the expression level of *BNP* even though it had already been stimulated by PE, indicating an exacerbated hypertrophic response. Next, cell apoptosis, a main pathogenesis of heart failure was assessed. The proportions of H9C2 cells that undergone early and late apoptosis induced by PE were significantly raised by *SVIL* siRNA interference (Fig 4D), and *SVIL* deficiency alone could increase the proportion of cells in the state of early apoptosis (Fig 4D**, left panel**). Furthermore, the level of cell viability was notably decreased owing to *SVIL* knockdown with PE exposure (Fig 4E). All these observations revealed that the loss of *SVIL* could play a role in the pathogenic process of heart failure. Subsequently, the effect of *SVIL* on other genes associated with cardiomyopathy was assessed by western blot analysis (Fig 4F). As expected, *SVIL* knockdown markedly decreased the levels of caveolin-3 and α-sarcoglycan which were previously identified in familial cardiomyopathy [64,65].

### Differentially expressed genes (DEG) and gene ontology (GO) pathway analysis

To reveal the molecular mechanism associated with *SVIL* gene, a DEG analysis was performed to identify gene expression changes between *SVIL* knockdown and control groups in H9C2 cells. A total of 435 DEGs (FDR ≤ 0.05 and fold-change ≥ 2.0) were detected, of which 187 genes were upregulated (higher expression in *SVIL* knockdown) and 248 genes were downregulated (S21 Table). According to the functional enrichment results, 17 GO pathways, including 13 biological process (BP) term, three cellular component (CC) term and a molecular function (MF) term were statistically significantly enriched in DEGs with rich factor >5. The most enriched GO pathway is response to muscle activity, with three upregulated genes (*PERM1*, *POSTN*, *PRKAA2*) and two downregulated genes (*AKAP5* and *MYOG*) involved (S22 Table). Among the 17 significantly enriched GO pathways, five were involved in the biological process and cellular component of heart muscle, including response to muscle activity, negative regulation of striated muscle cell differentiation, neuromuscular junction, myofibril assembly and regulation of myotube differentiation (Fig 4G and S22 Table).

## Discussion

In the present study, we identified a region associated with all-cause HF and another specific to NICM and replicated all 106 previously identified regions in the East Asian population. Inclusion of China PEACE 5p-HF/ChinaHEART, BBJ, and UKB adds statistical power and diversity, especially for the East Asian populations, which are often underrepresented in genetic studies. Our findings contribute to the growing body of genetic and bioinformatics evidence underscoring the roles of left ventricular remodeling and sarcomere structure as important drivers of HF pathophysiology in the East Asian populations.

Through bioinformatic analyses and literature review, our results highlighted the functional roles of several potential causal genes related to sarcomere structure based on the bioinformatic analyses and literature review. Notably, the 2q31.2 locus tagged by the lead SNP rs17076 harbored *TTN*, a gene that encodes for the largest-known human protein, at which rare truncating mutations were detected in 25% of familial DCM and 18% of idiopathic DCM [66]. TTN has a vital role in the regulation of sarcomere assembly and the preservation of diastolic and systolic function [67]. Since the lead SNP in our GWAS is intronic, we assessed whether it could affect expression or splicing of the *TTN* gene using the GTEx Project. Interestingly, the HF risk allele C was associated with increased intron-excision ratio of *TTN* and this splicing effect was stronger than its expression consequence in sarcomere relevant tissues including skeleton muscle and heart. While the role of truncation mutations of *TTN* in cardiomyopathy is accepted, it is likely the mutant allele would produce alterations in titin isoform composition or expression level with abnormal properties that contributes to HF.

When gene expression data were used to prioritize candidate causal genes, the *MTSS1* (metastasis suppressor protein 1) gene emerged as a strong candidate causal gene for all-cause HF. MTSS1 interacts with actin cytoskeleton and cell membrane to regulate cell structure and intercellular junctions [68]. When investigating the *cis*-associations with gene expression in the eQTLgen Consortium and GTEx database, the lead variant rs12542527 could be linked to HF via a gene regulatory effect on *MTSS1* expression. In this regard, previous studies have shown the reduction in *MTSS1* may promote favorable LV remodeling in both mice [69] and humans [70,71].

In our analysis, the lead SNP (rs139616510; C > T) at *MYBPC3* locus yielded the strongest effect size for HF (OR = 1.73) out of all risk variants. Rs139616510 is in high LD (r ^2^ = 1) with the missense variant rs573916965, which leads to a rare (~0.5% frequency in East Asian populations, and ~0.006% in the UKB) Glu334Lys mutation in exon 12 of *MYBPC3* encoding the cardiac myosin-binding protein C (cMyBP-C). Approximately half of *MYBPC3* mutations give rise to truncated protein products of cMyBP-C in patients with HCM [72], which is the primary structural protein of the heart muscle, and interacts with actin, myosin, and titin to maintain the integrity of the sarcomeric layer [73]. Reduced cMyBP-C levels (protein haploinsufficiency) can occur either due to the mutant not being created or because of it breaking down rapidly [74]. Additionally, if the mutant protein with the shortened amino acid is produced and becomes dominant, it may operate as a “poison peptide” by exerting a dominant-negative influence and modifying the structure and function of the sarcomere [75]. Identified first in Japanese HCM patients [76], Glu334Lys mutation might also destabilize its protein through the ubiquitin-proteasome system (UPS) [76], and contribute to cardiac dysfunction by altering the protein levels of Ca^2+^ handling proteins and action potential durations [77]. This is the first time Glu334Lys mutation being identified in large-scale genetic analysis for HF in the East Asian populations.

Another important aspect of our study is the *in vitro* functional validation of *SVIL* as potential candidate causal gene at the chromosome 10q11.23 locus, which may point to a role of the pathways involving myocardial structure and heart development in the development of NICM. In the China PEACE 5p-HF/ChinaHEART, the G allele of the lead SNP rs2182400 at the *SVIL* locus increased the risk of NICM with stronger magnitude (OR = 1.14, P = 6.4 × 10^-4^) than with all-cause HF (OR = 1.09, P = 4.1 × 10^-3^), indicating the association signal is not primarily mediated via ischemic conditions induced by CAD and MI. This hypothesis is further validated in the phenome-wide association approach (PheWAS) as there is no associations between rs2182400 and common heart failure risk factors including CAD, MI, AF, BP, BMI, lipid and T2D. Consistent with our observation, rs2182400 was only nominally associated with increased risk of all-cause HF (OR = 1.02, P = 2.2 × 10^-3^) from previous large-scale GWAS in European population [12]. Unlike the loss of function variant, our observation suggest that a regulatory mediator is most likely involved, as the G allele of rs2182400 would potentially increase splicing and decrease *SVIL* gene expression level. Further work is needed to clarify potential regulatory mechanisms at this locus (including antisense transcripts *SVIL-AS1*) to provide a forward-looking perspective.

In cardiac and skeletal muscles, the most abundantly expressed isoform is archvillin-the largest (∼250 kDa), differentially spliced isoform 2 of supervillin [78,79]. It is worth noting that loss of supervillin has been reported in four myopathy patients presented with myofibrillar structural abnormalities [61]. As a binding partner to major sarcolemma-associated proteins, supervillin is known to function as part of a system keeping the structural integrity of muscle fibres [80]. Loss of supervillin leads to impaired myofibrillar cytoskeleton and formation of autophagic vacuoles [61], which is responsible for cardiomyopathy with altered proteostasis and myofibrillar disorganization. Furthermore, the important role of supervillin in maintaining normal cardiac function was further validated with animal models. Previous study confirmed a significant decrease in atrial conduction velocity following morpholino knockdown of *SVIL* in a zebrafish model [81].

Multiple data from our functional and bioinformatics analyses support the mechanistic roles of *SVIL* in the pathogenesis of NICM. First, *SVIL* deficiency aggravated cardiomyocyte hypertrophy, apoptosis and impaired cell viability in phenylephrine (PE)-treated H9C2 cells. In addition, increased levels of HF markers were observed in *SVIL* knockdown H9C2 cell. Second, the sarcomere related protein (α-sarcoglycan and caveolin-3) was reduced when the expression of *SVIL* was inhibited. Caveolin-3 (Cav-3), a component of caveolae, is expressed in cardiac, skeletal, and smooth muscles and interacts with angiotensin II type 1 receptor that plays an important role in cardiac hypertrophy [82]. Specifically, previous studies have shown that the loss of caveolin-3 causes cardiac hypertrophy via hyperactivation of p42/44 MAPK cascade [83] and the dysregulation of muscle calcium homeostasis [84]. Caveolin-3 is also associated with the α-subunit of the Nav1.5 ion channel, which has been suggested as the mechanism of *CAV3*-associated long QT syndrome or sudden infant death syndrome [85]. Caveolae are involved in not only cellular metabolic regulation through calcium signaling but also glucose and lipid metabolism [86]. Caveolae provide an important contribution to the cell surface area of adipocytes and may facilitate lipid fatty acid trafficking [87]. CAV3 is a regulator of lipid uptake in the heart, loss of CAV3 interferes with downstream insulin signaling and lipid uptake [88]. And it has been suggested that caveolae may regulate cellular fatty acid uptake by FAT/CD36 [89]. Among the 17 significantly enriched GO pathways on DEGs by *SVIL* knockdown, CAV3 were involved in the biological process and cellular component of heart muscle, including negative regulation of striated muscle cell differentiation, neuromuscular junction, myofibril assembly and regulation of myotube differentiation. On the other hand, sarcoglycans play a vital role in preventing muscle damage during muscle contraction and all four sarcoglycans (α-, β-, γ-, and δ-sarcoglycan, respectively) must be present to form a functional sarcoglycan complex [90]. Loss of the α-sarcoglycan (α-SG) protein leads to a progressive muscular dystrophy with deteriorating muscle function, including dystrophic features such as muscle necrosis and fibrosis, elevated serum creatine kinase (CK), and reduction in the generation of absolute muscle force and locomotor activity [91]. It should be noted that the caveolin-3 and α-sarcoglycan results remain hypothesis-generating and need further validation. Third, GO pathway analysis on DEGs by *SVIL* knockdown revealed that the function of *SVIL* might be mediated through pathways relevant to regulation and differentiation of heart muscle. A better understanding of *SVIL*’s biological functions in heart and its link with caveolin-3 and α-sarcoglycan will be needed to determine its role in the development of NICM.

To understand the genetic basis of HF and NICM in the East Asian population, we have compared the difference between allele frequencies and effect sizes of the identified loci across different populations. The effect of *SVIL* variant rs2182400 on DCM was comparable across different populations despite a lower EAF in Europeans (EAF = 0.24, OR=1.10) [17,38] than the China study (EAF = 0.53, OR=1.14). It should be noted that an association between the same *SVIL* variant and HCM has been reported in European populations [40]. Since the current study did not include HCM cases, we cannot conclude if this effect is specific to the European population. Moreover, *SVIL* variant rs2182400 showed strong evidence of association with reduced LVEF and increased LVESV in the UKB dataset which did not include HF and MI patients [37], and these effects were consistent with increased NICM risk in the China study. Sensitivity analysis and the multi-trait conditional analysis suggest the possibility of a role of *SVIL* on NICM were partially explained by its established influence on LVEF and HCM. However, definitive proof of this hypothesis will require further study. In addition, *SVIL* has been identified as one of the strongest GWAS signals for the descending aorta [41] and right ventricular ejection fraction [42] in the UKB individuals. However, their associations with NICM were not significant in the China study. As for the *MYBPC3* locus, the low-frequency East-Asian enriched coding variant rs139616510 only yields a MAF of ~0.006% in the UKB, and the European enriched rare variants associated with HCM and its subtypes [40] all have a MAF of 0 in the China study. On the other hand, the common variant (rs11570078) associated with DCM_MTAG [39] (EAF = 0.87) was not associated with HF in the China study (EAF = 0.95, P = 0.262). Taken together, the genetics effect of *SVIL* seemed to be comparable across ancestries, and the discrepancy at *MYBPC3* locus might be driven by the differences in allele frequency and population-specific effects.

Males and females with HF showed different characteristics across the EF spectrum [92]. Sex-related differences in HF may involve baseline characteristics, treatment, and prognosis. For example,females were older and more likely to have hypertension and kidney disease but less likely to have diabetes and ischemic heart disease. Females were more likely to use beta-blockers and digoxin but less likely to receive HF device therapy. Mortality risk was significantly lower in females regardless of EF. The main sex-related differences in prognostic predictors concerned diabetes in HFrEF and anemia in HFmrEF [92]. However, few GWAS have examined sex differences in the genetic architecture of HF specifically. *MYBPC3* locus was identified in the male-specific GWAS and showed stronger effect in males than in females. One explanation might be the higher expression of proteins associated with apoptosis (i.e., *Fas* and *TNFR1*) in males compared to females, whose level results in lower level of LV recovery for patients with recent-onset cardiomyopathy [93]. Previous studies have also shown that sex affects the penetrance and pathology of genetic cardiomyopathies with male being more severely affected [94]. Here, due to the unavailability of sex-stratified GWAS results in the UKB, we did not perform sex-stratified meta-analyses for NICM. Whether sex-specific genetic associations exist will be an important area of future study.

While our results point to distinct genetic determinants of HF and NICM, certain limitations should be taken into considerations. First, the China PEACE 5p-HF/ChinaHEART is primarily Chinese population, whereas the majority of subjects in the UKB were of European ancestry and possibly a confounding factor for the NICM meta-analysis. There also exist asymmetry in the population representation due to limited NICM data in non-European cohorts. The discrepancy in case definition could meaningfully affect GWAS results and may partially explain differences in signal strength or loci identified across cohorts. The UKB defined NICM cases more specifically as individuals with evidence of left ventricular systolic dysfunction in the absence of prior CAD. As a result, the NICM group in the China PEACE 5p-HF cohort may include both HFrEF and HFpEF cases, introducing phenotypic heterogeneity. In the sensitivity analyses for NICM in the China PEACE 5p-HF cohort, the signal of lead SNP rs2182400 at *SVIL* appears enriched in HFrEF versus HFpEF. We noted that the discrepancy in case definition has biased the results towards the null. Second, the use of H9C2 cells are embryonic rat cardiomyoblasts that do not fully recapitulate adult human cardiomyocyte biology. Additionally, phenylephrine (PE) is a nonspecific hypertrophic stimulus, which may limit the generalizability of the *in vitro* findings. Moreover, there is a power limitation, particularly in sex-stratified analyses (only 1,457 HF cases and 7,001 controls in the female HF GWAS). With this sample size, assuming the global prevalence of HF is 2% in the total population (range between 1% and 3% [95]), approximately 80% power could be obtained when the variants with MAF ranging from 0.1 to 0.5 have corresponding effect sizes of 1.20 to 1.12, respectively. In addition, the low MAF of the lead SNP rs139616510 at *MYBPC3* (~0.5%) poses challenges for replication and increases the risk of winner’s curse. Ideally, replication in another East Asian dataset would be ideal, but it is not possible in the current study. Lastly, the number of novel findings is limited, as *MYBPC3* and *SVIL* loci have been identified in recent HCM and DCM GWAS. Nevertheless, the breadth of functional work on *SVIL* makes an interesting addition to the literature.

In summary, large-scale genetics analyses identified a low-frequency East-Asian enriched coding variant near *MYBPC3* locus for all-cause HF and a locus for a more restrictive NICM phenotype in multi-ancestry populations. Follow-up analyses demonstrated male-specific HF association at *MYBPC3* locus that warrants further replication in another datasets, and functionally validated *SVIL* as one candidate causal gene that are associated with subclinical left ventricular dysfunction. These results tackle the lack of population diversity in GWAS, and provide opportunities for further exploration of the biological mechanisms underlying the pathogenesis of HF and left ventricular remodeling.

## Supporting information

S1 Fig Results of GWAS analysis for HF in the China PEACE 5p-HF/ChinaHEART. **(A)** A Manhattan plot shows no genome-wide significantly associated SNPs with MAF > 1% probably due to limited power. The GWAS analysis included 3,972 HF cases and 11,171 controls. Genome-wide thresholds for significant (P = 5.0x10^-8^) and suggestive (P = 5.0x10^-6^) association are indicated by the horizontal red and dark blue lines, respectively. P-values are truncated at -log_10_(P)=8. **(B)** A quantile-quantile plot shows the observed versus the expected P-values from the association analyses for HF in the China PEACE 5p-HF/ChinaHEART. The genomic control factor (λ) in the China PEACE 5p-HF/ChinaHEART result was 1.135 and the LD Score intercept was 1.104 (SE = 0.0063).
(TIFF)

S2 Fig Quantile-quantile plots for results of GWAS meta-analysis for HF with the China PEACE 5p-HF/ChinaHEART and Biobank Japan. The observed versus the expected P-values from the fixed effect meta-analysis are shown. The meta-analysis for all-cause HF included a total of 13,385 HF cases and 214,211 controls from the China PEACE 5p-HF/ChinaHEART (3,972 HF cases and 11,171 controls) and the Biobank Japan datasets (9,413 HF cases and 203,040 controls) with 5,887,003 SNPs common to both datasets. The genomic control factor (λ) in the meta-analysis was 1.103 and the LD Score intercept was 1.059 (SE = 0.0073), suggesting that any inflation of test statistics was more likely due to many small genetic effects rather than population structure.
(TIFF)

S3 Fig Regional plot of the *MYBPC3* locus identified for HF in GWAS meta-analyses with the China PEACE 5p-HF/ChinaHEART and the Biobank Japan. The *MYBPC3* locus is centered on the lead SNP (purple diamond) and the genes in the interval are indicated in the bottom panel. The degree of linkage disequilibrium (LD) between the lead SNP and other variants is shown as r ^2^ values according to the color-coded legend in the box.
(TIFF)

S4 Fig Male-specific GWAS meta-analysis for HF with the China PEACE 5p-HF/ChinaHEART and Biobank Japan. A quantile-quantile plot shows the observed versus the expected P-values from the male-specific GWAS meta-analysis for HF. The genomic control factor (λ) in the meta-analysis was 1.071 and the LD Score intercept was 1.051 (SE = 0.0074), suggesting that any inflation of test statistics was more likely due to many small genetic effects rather than population structure.
(TIFF)

S5 Fig Female-specific GWAS meta-analysis for HF with the China PEACE 5p-HF/ChinaHEART and Biobank Japan. A quantile-quantile plot shows the observed versus the expected P-values from the female-specific GWAS meta-analysis for HF. The genomic control factor (λ) in the meta-analysis was 1.011 and the LD Score intercept was 0.993 (SE = 0.0064).
(TIFF)

S6 Fig Quantile-quantile plots for results of GWAS meta-analysis for NICM with the China PEACE 5p-HF/ChinaHEART and the UK Biobank. The observed versus the expected P-values from the fixed effect meta-analysis are shown. The meta-analysis for NICM included a total of 3,603 NICM cases and 399,497 controls from the China PEACE 5p-HF/ChinaHEART (1,787 NICM cases and 11,171 controls) and the UK Biobank (1,816 NICM cases and 388,326 controls) with 4,567,081 SNPs common to both datasets. The genomic control factor (λ) in the meta-analysis was 1.065 and the LD Score intercept was 1.064 (SE = 0.0088).
(TIFF)

S7 Fig Regional plot of the *SVIL* locus identified for NICM in GWAS meta-analyses with the China PEACE 5p-HF/ChinaHEART and the UK Biobank. The *SVIL* locus is centered on the lead SNP (purple diamond) and the genes in the interval are indicated in the bottom panel. The degree of linkage disequilibrium (LD) between the lead SNP and other variants is shown as r ^2^ values according to the color-coded legend in the box.
(TIFF)

S8 Fig Expression level of *SVIL* in siRNA-mediated knockdown in H9C2 cells. The mRNA level of *SVIL* was detected by RT-qPCR in H9C2 cells transfected with siRNA. N = 4.
(TIFF)

S1 Table Description of datasets used for GWAS meta-analysis for Heart failure (HF) and nonischemic cardiomyopathy (NICM). (XLSX)

S2 Table Sample quality control procedures performed for the China PEACE 5p-HF/ChinaHEART. (XLSX)

S3 Table SNP quality control procedures performed for the China PEACE 5p-HF/ChinaHEART. (XLSX)

S4 Table Study-level participant characteristics. (XLSX)

S5 Table 6 loci identified for heart failure in meta-analysis of the China PEACE 5p-HF/ChinaHEART and Biobank Japan. (XLSX)

S6 Table Replication of previously reported loci for heart failure. (XLSX)

S7 Table 3 loci identified for heart failure in the male-specific meta-analysis of the China PEACE 5p-HF/ChinaHEART and Biobank Japan. (XLSX)

S8 Table Combined and sex-stratified results for 6 loci identified for heart failure in meta-analysis of the China PEACE 5p-HF/ChinaHEART and Biobank Japan. (XLSX)

S9 Table 3 loci identified for non-ischemic cardiomyopathy (NICM) in meta-analysis of the China PEACE 5p-HF/ChinaHEART and the UK Biobank. (XLSX)

S10 Table Association of 4 HF Loci with HF Risk Factors in literature. (XLSX)

S11 Table Association of 4 HF Loci with cardiac magnetic resonance imaging (MRI)-derived left ventricular measure in the UK Biobank. (XLSX)

S12 Table Comparison between effect estimates of *SVIL* from the present study and previous studies in the DCM, HCM and MRI-derived cardiac function GWAS. (XLSX)

S13 Table Comparison between effect estimates of *MYBPC3* from the present study and previous studies in the DCM, and HCM GWAS. (XLSX)

S14 Table Results of Data-driven Expression-Prioritized Integration for Complex Traits (DEPICT) gene set enrichment analysis with SNPs significantly (FDR < 0.05) associated with heart failure in GWAS meta-analysis of the China PEACE 5p-HF/ChinaHEART and Biobank Japan. (XLSX)

S15 Table Results of Data-driven Expression-Prioritized Integration for Complex Traits (DEPICT) tissue enrichment analysis with SNPs significantly (FDR < 0.05) associated with heart failure in GWAS meta-analysis of the China PEACE 5p-HF/ChinaHEART and Biobank Japan. (XLSX)

S16 Table Sensitivity analysis for *SVIL* locus in the China PEACE 5p-HF/ChinaHEART. (XLSX)

S17 Table Pairwise genetic correlation between HF/NICM and 10 GWAS traits estimated using LDSC. (XLSX)

S18 Table Conditional GWAS analyses for *SVIL* locus. (XLSX)

S19 Table Multi-tissue expression quantitative trait Locus (eQTLs) at 4 HF loci. (XLSX)

S20 Table Multi-tissue splice quantitative trait locus (sQTLs) at 4 HF loci from the GTEx project (version 8). (XLSX)

S21 Table Differentially expressed genes (DEG) between *SVIL* knock down and controls in H9C2 cells. (XLSX)

S22 Table Significantly enriched pathways from Gene Ontology (GO) analysis. (XLSX)
