# Supplementary figures and images for: Genome-wide analysis identifies susceptibility loci for heart failure and nonischemic cardiomyopathy subtype in the East Asian populations
Source: PLoS Genet. 2025 Oct 27;21(10):e1011897. doi: 10.1371/journal.pgen.1011897 (PMC12558498; doi:10.1371/journal.pgen.1011897)

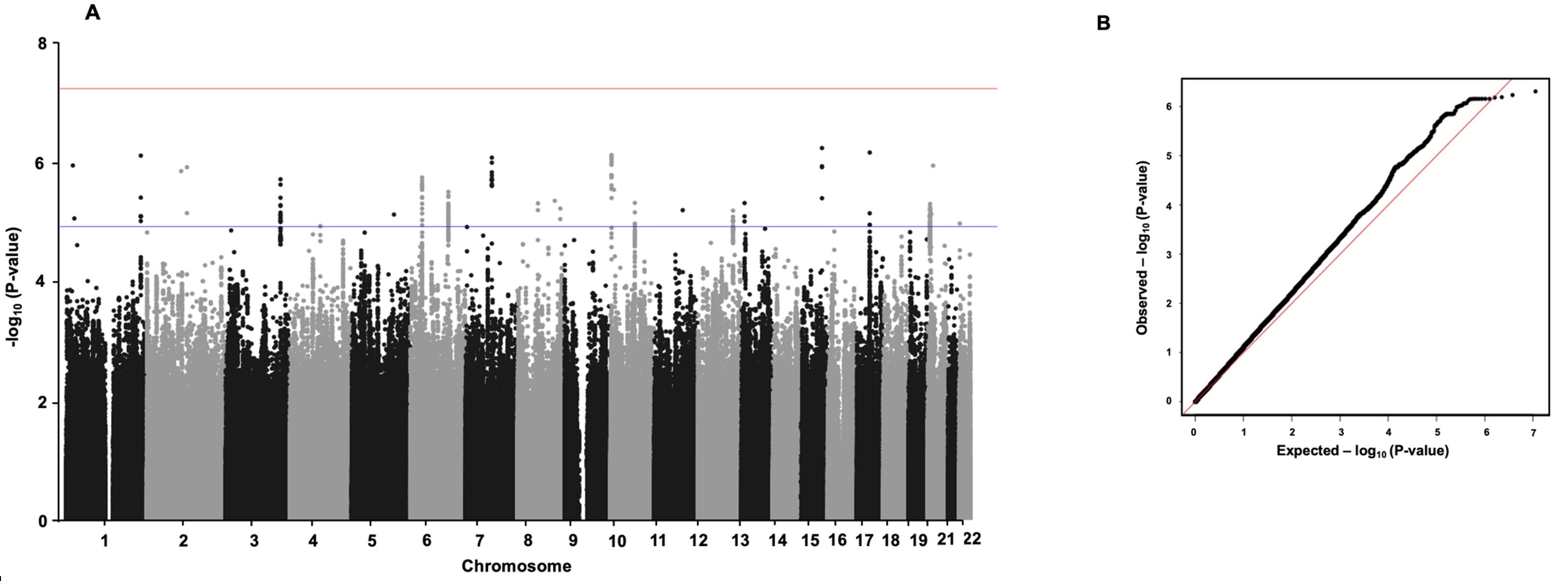

Supplement: S1 Fig — (A) A Manhattan plot shows no genome-wide significantly associated SNPs with MAF > 1% probably due to limited power. The GWAS analysis included 3,972 HF cases and 11,171 controls. Genome-wide thresholds for significant (P = 5.0x10^-8^) and suggestive (P = 5.0x10^-6^) association are indicated by the horizontal red and dark blue lines, respectively. P-values are truncated at -log10(P)=8. (B) A quantile-quantile plot shows the observed versus the expected P-values from the association analyses for HF in the China PEACE 5p-HF/ChinaHEART. The genomic control factor (λ) in the China PEACE 5p-HF/ChinaHEART result was 1.135 and the LD Score intercept was 1.104 (SE = 0.0063). (TIFF) [file pgen.1011897.s001.tiff]

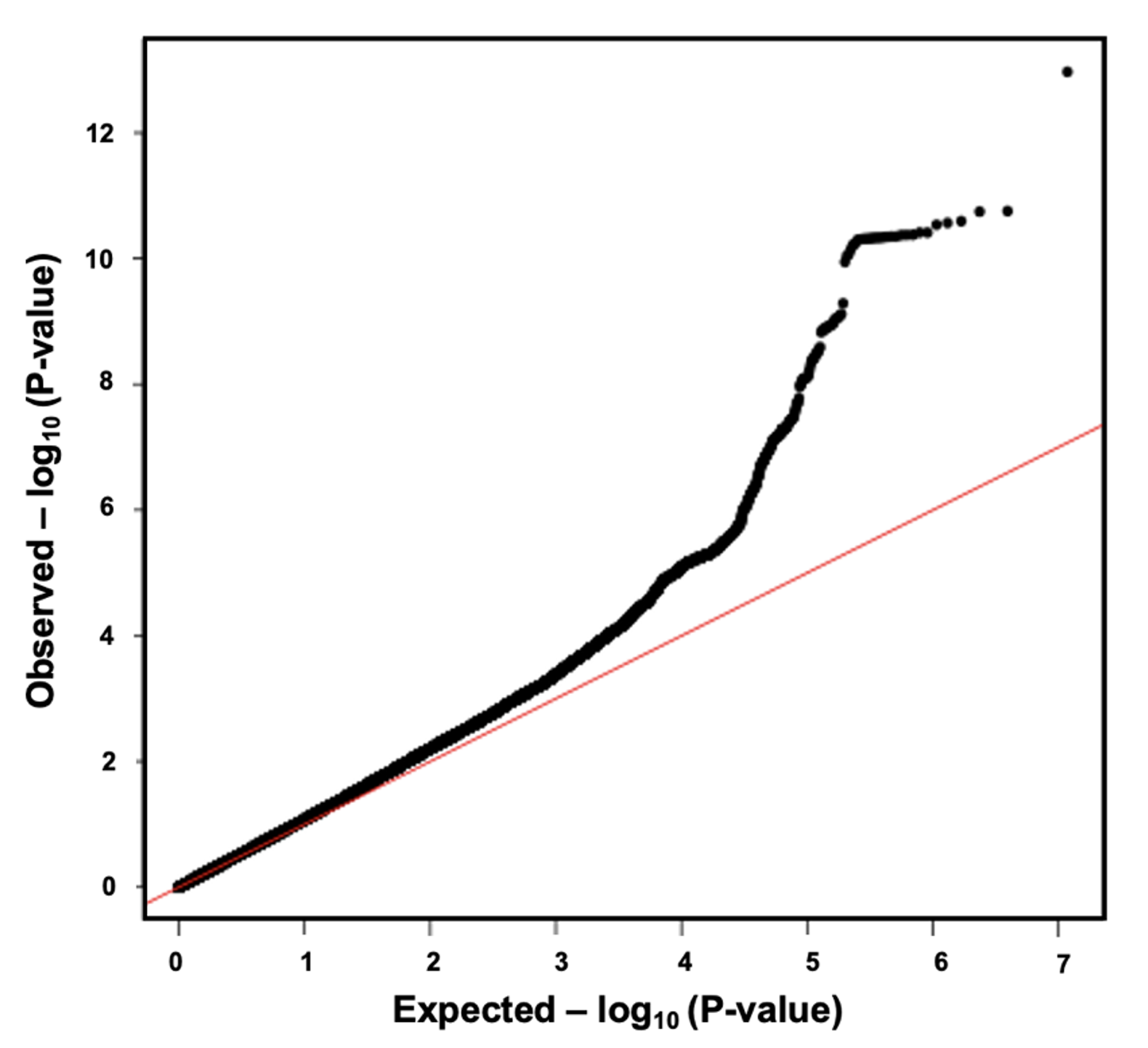

Supplement: S2 Fig — The observed versus the expected P-values from the fixed effect meta-analysis are shown. The meta-analysis for all-cause HF included a total of 13,385 HF cases and 214,211 controls from the China PEACE 5p-HF/ChinaHEART (3,972 HF cases and 11,171 controls) and the Biobank Japan datasets (9,413 HF cases and 203,040 controls) with 5,887,003 SNPs common to both datasets. The genomic control factor (λ) in the meta-analysis was 1.103 and the LD Score intercept was 1.059 (SE = 0.0073), suggesting that any inflation of test statistics was more likely due to many small genetic effects rather than population structure. (TIFF) [file pgen.1011897.s002.tiff]

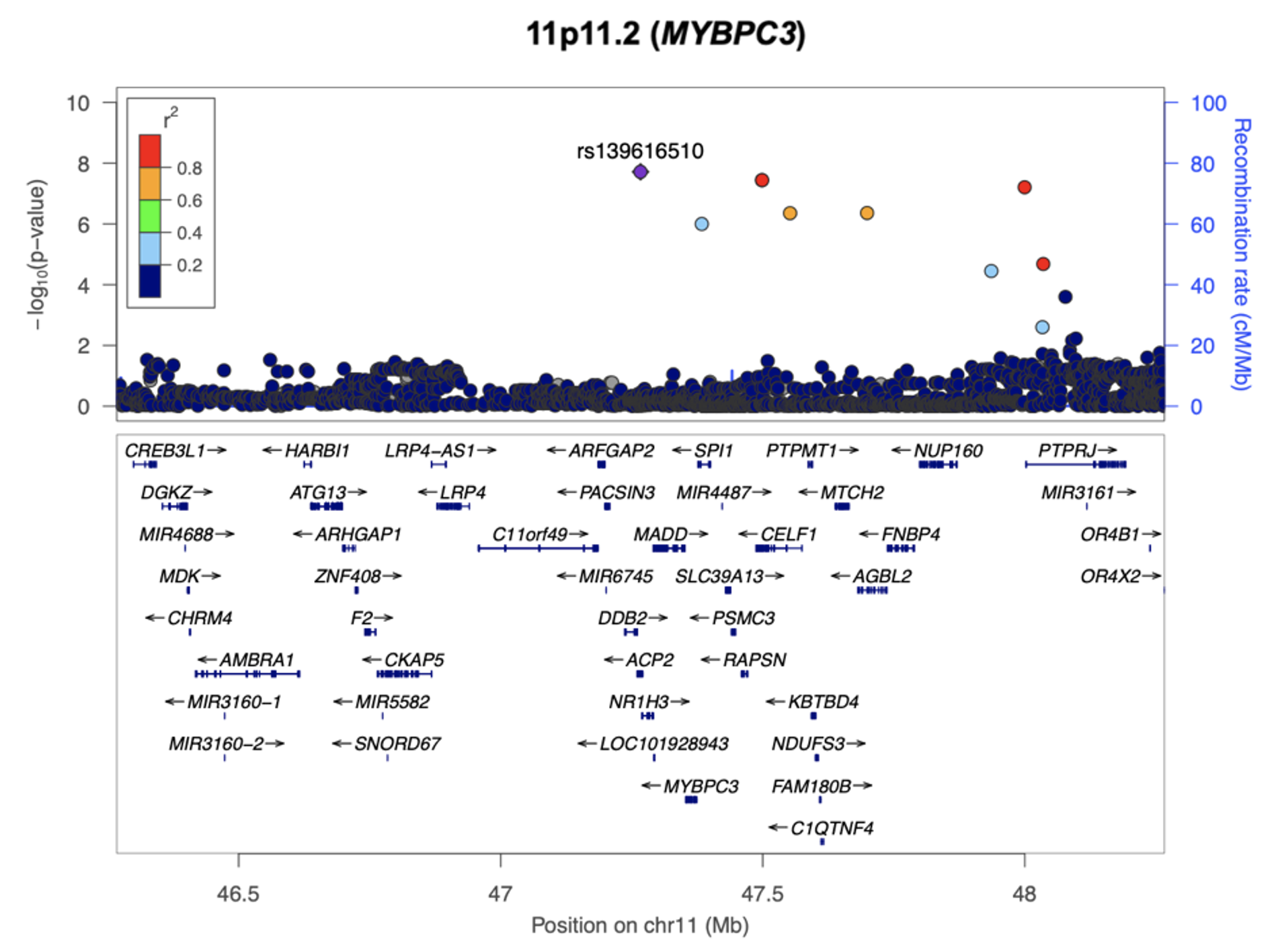

Supplement: S3 Fig — The MYBPC3 locus is centered on the lead SNP (purple diamond) and the genes in the interval are indicated in the bottom panel. The degree of linkage disequilibrium (LD) between the lead SNP and other variants is shown as r 2 values according to the color-coded legend in the box. (TIFF) [file pgen.1011897.s003.tiff]

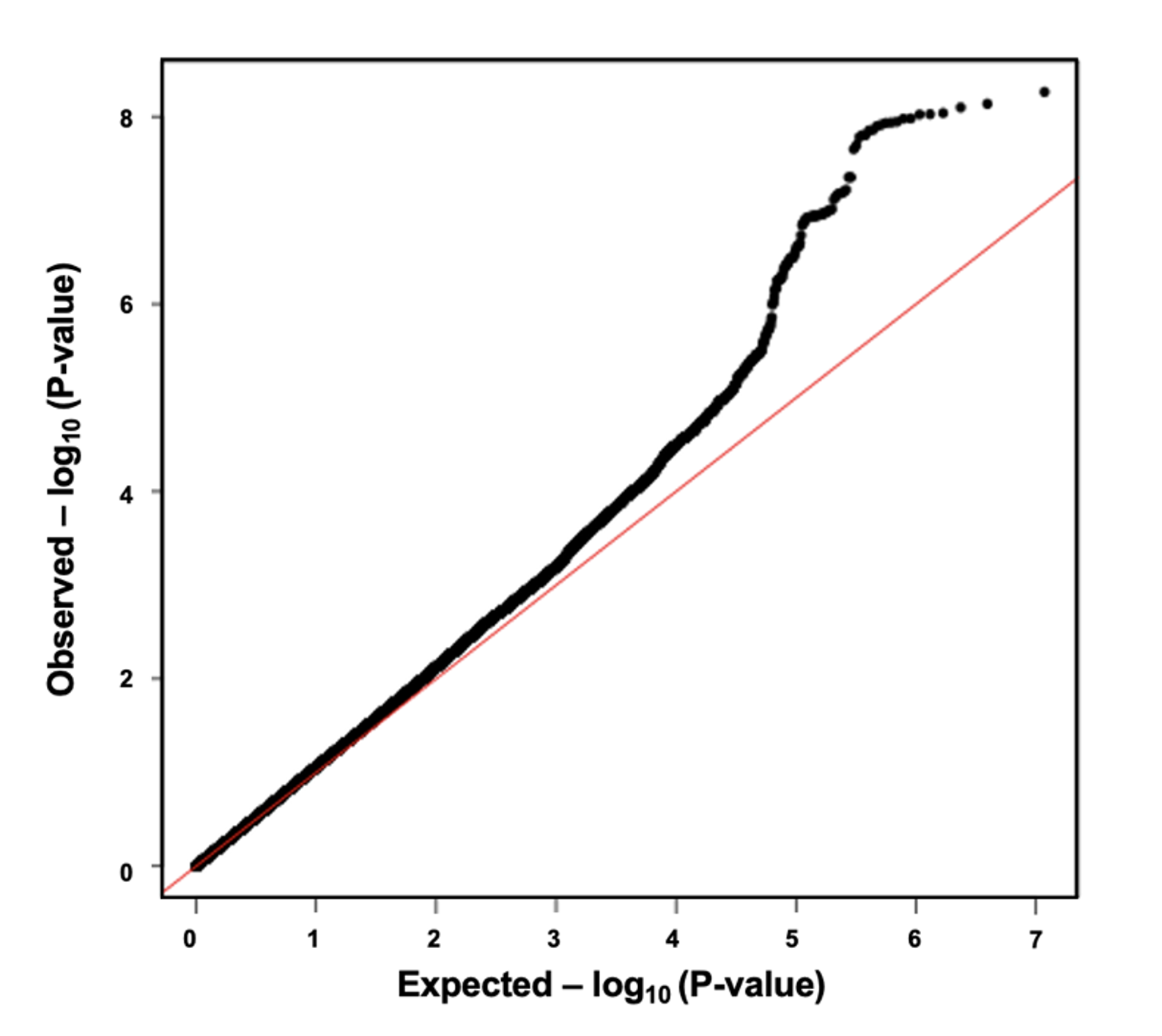

Supplement: S4 Fig — A quantile-quantile plot shows the observed versus the expected P-values from the male-specific GWAS meta-analysis for HF. The genomic control factor (λ) in the meta-analysis was 1.071 and the LD Score intercept was 1.051 (SE = 0.0074), suggesting that any inflation of test statistics was more likely due to many small genetic effects rather than population structure. (TIFF) [file pgen.1011897.s004.tiff]

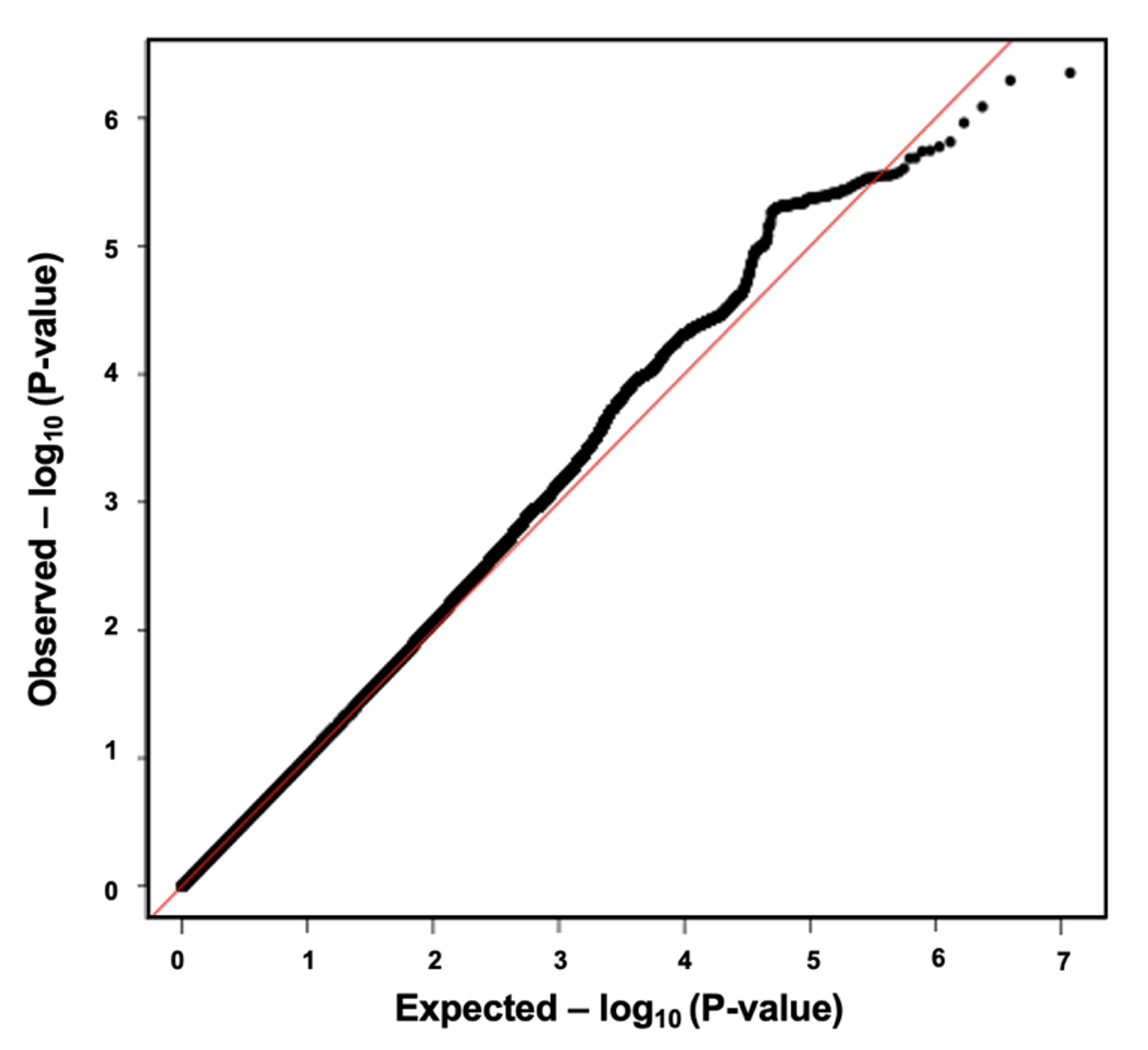

Supplement: S5 Fig — A quantile-quantile plot shows the observed versus the expected P-values from the female-specific GWAS meta-analysis for HF. The genomic control factor (λ) in the meta-analysis was 1.011 and the LD Score intercept was 0.993 (SE = 0.0064). (TIFF) [file pgen.1011897.s005.tiff]

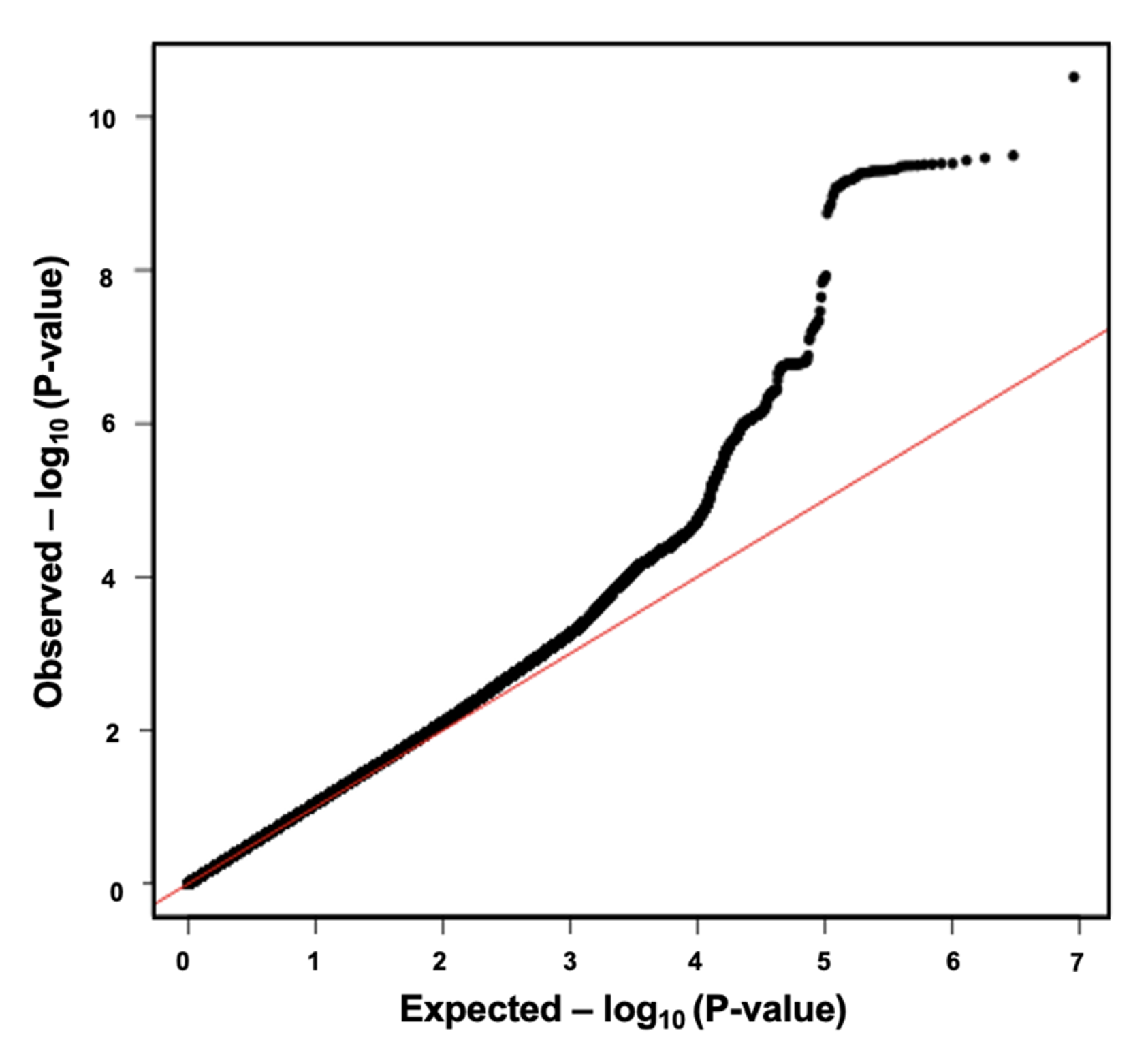

Supplement: S6 Fig — The observed versus the expected P-values from the fixed effect meta-analysis are shown. The meta-analysis for NICM included a total of 3,603 NICM cases and 399,497 controls from the China PEACE 5p-HF/ChinaHEART (1,787 NICM cases and 11,171 controls) and the UK Biobank (1,816 NICM cases and 388,326 controls) with 4,567,081 SNPs common to both datasets. The genomic control factor (λ) in the meta-analysis was 1.065 and the LD Score intercept was 1.064 (SE = 0.0088). (TIFF) [file pgen.1011897.s006.tiff]

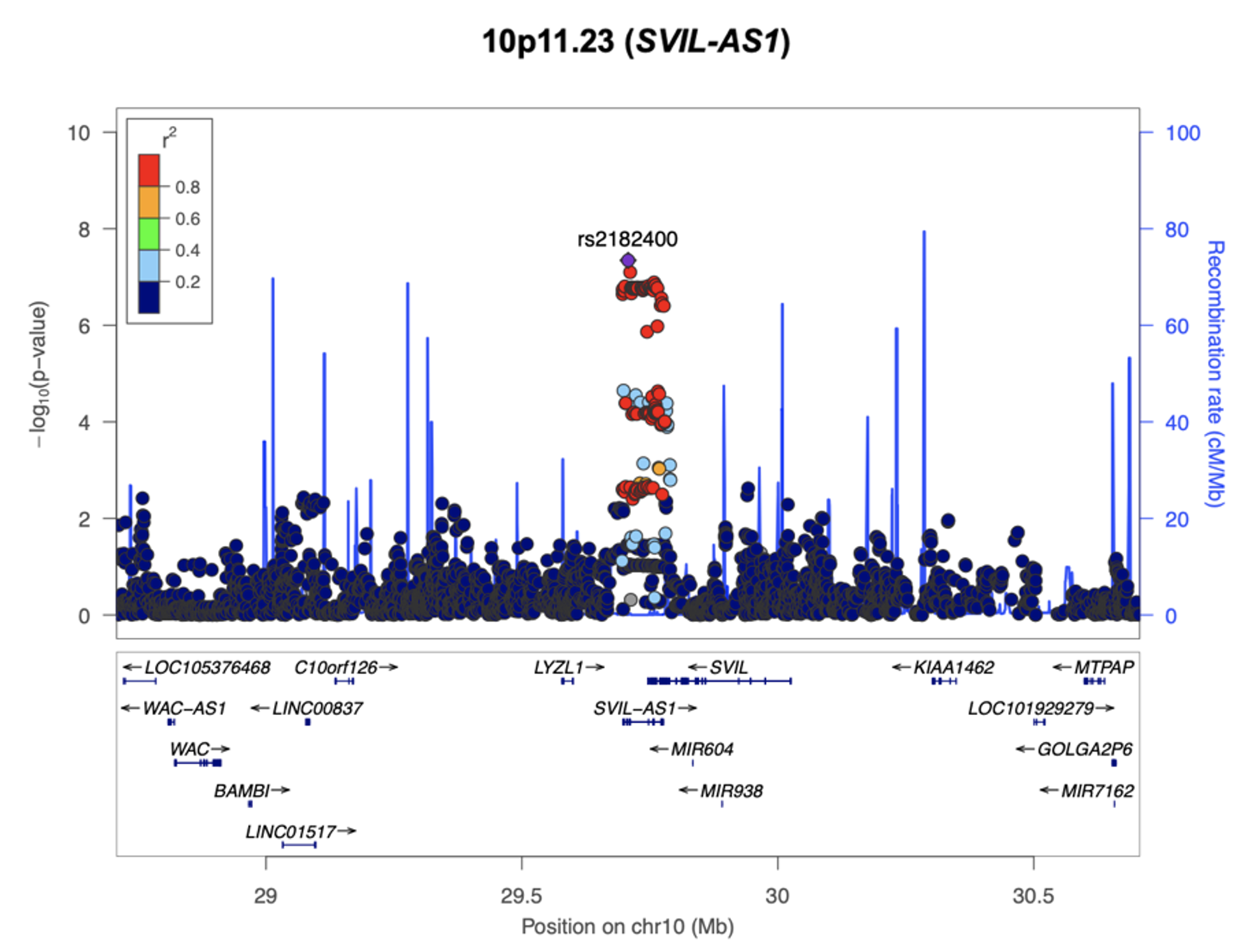

Supplement: S7 Fig — The SVIL locus is centered on the lead SNP (purple diamond) and the genes in the interval are indicated in the bottom panel. The degree of linkage disequilibrium (LD) between the lead SNP and other variants is shown as r 2 values according to the color-coded legend in the box. (TIFF) [file pgen.1011897.s007.tiff]

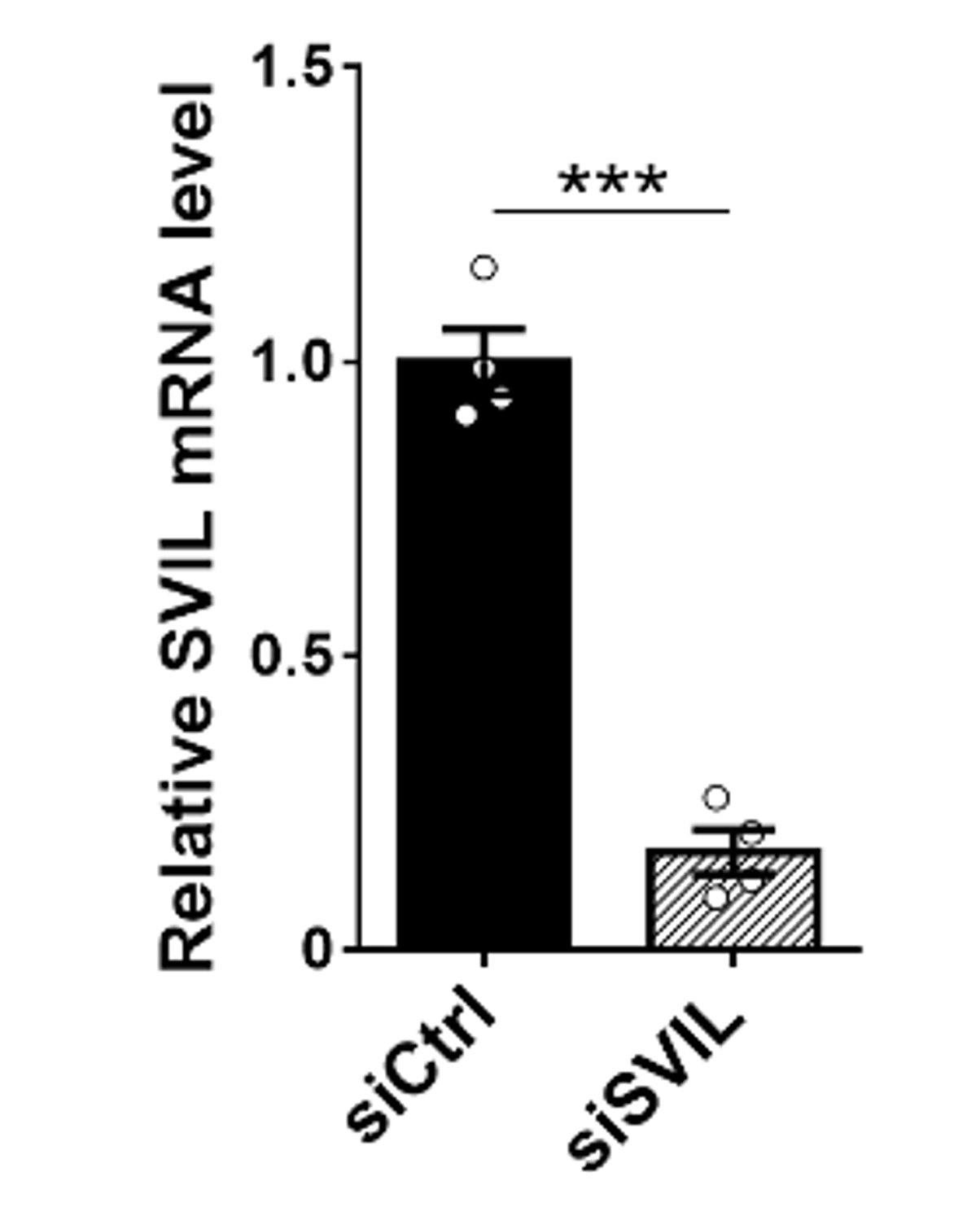

Supplement: S8 Fig — The mRNA level of SVIL was detected by RT-qPCR in H9C2 cells transfected with siRNA. N = 4. (TIFF) [file pgen.1011897.s008.tiff]
